# Fgr contributes to hemorrhage-induced thalamic pain by activating NF-**κ**B/ERK1/2 pathways

**DOI:** 10.1172/jci.insight.139987

**Published:** 2020-10-15

**Authors:** Tianfeng Huang, Ganglan Fu, Ju Gao, Yang Zhang, Weihua Cai, Shaogen Wu, Shushan Jia, Shangzhou Xia, Thomas Bachmann, Alex Bekker, Yuan-Xiang Tao

**Affiliations:** 1 Department of Anesthesiology;; 2 Department of Pharmacology, Physiology & Neuroscience; and; 3 Department of Cell Biology & Molecular Medicine, New Jersey Medical School, Rutgers, The State University of New Jersey, Newark, New Jersey, USA.

**Keywords:** Neuroscience, Pain

## Abstract

Thalamic pain, a type of central poststroke pain, frequently occurs following ischemia/hemorrhage in the thalamus. Current treatment of this disorder is often ineffective, at least in part due to largely unknown mechanisms that underlie thalamic pain genesis. Here, we report that hemorrhage caused by microinjection of type IV collagenase or autologous whole blood into unilateral ventral posterior lateral nucleus and ventral posterior medial nucleus of the thalamus increased the expression of Fgr, a member of the Src family nonreceptor tyrosine kinases, at both mRNA and protein levels in thalamic microglia. Pharmacological inhibition or genetic knockdown of thalamic Fgr attenuated the hemorrhage-induced thalamic injury on the ipsilateral side and the development and maintenance of mechanical, heat, and cold pain hypersensitivities on the contralateral side. Mechanistically, the increased Fgr participated in hemorrhage-induced microglial activation and subsequent production of TNF-α likely through activation of both NF-κB and ERK1/2 pathways in thalamic microglia. Our findings suggest that Fgr is a key player in thalamic pain and a potential target for the therapeutic management of this disorder.

## Abstract 2

Fgr, a member of the Src-family nonreceptor tyrosine kinases, promotes hemorrhage-induced thalamic pain through both NF-κB and ERK1/2-triggered TNF-α production in thamalic microglia.

## Graphical abstract

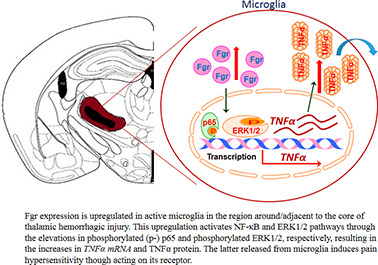

## Introduction

Central poststroke pain (CPSP), a chronic neuropathic pain syndrome, is often induced by damage and/or dysfunction of the central nervous system following stroke. Approximately 8%–14% of stroke victims suffer from CPSP, especially when a hemorrhagic stroke occurs in the thalamic area (1). Patients with CPSP commonly experience long-term hyperalgesia, allodynia, spontaneous pain, and other sensory deficits, in some cases for the rest of their lives. These pain hypersensitivities impair the patients’ daily activities, consequently undermining their quality of life and rehabilitation process (2, 3). Persistent CPSP and associated dysfunction (e.g., loss of sleep, social interaction, and ability to work) also lead to further mental health disorders, in particular depression and anxiety (4). However, current treatment for CPSP is very limited (5, 6). A systematic review of randomized controlled trials showed that opioids, antidepressants, and anticonvulsants not only had minimal or no effect on CPSP relief but also led to numerous troublesome side effects (7). Thus, understanding the mechanisms underlying CPSP may open a new avenue for therapeutic management of this disorder.

Accumulating evidence has revealed that direct ischemic or hemorrhagic injury to the brain leads to robust central inflammatory responses involving the resident immunologically active glial cells (8). In a mouse model of CPSP induced by injecting collagenase into the ventral posterior lateral nucleus (VPL) of the thalamus, prolonged microglial activation helped maintain mechanical allodynia and heat hyperalgesia, as intraperitoneal injection of minocycline, a microglial inhibitor, markedly ameliorated neuropathic pain-like behaviors (9). Microglial depletion effectively prevents mechanical allodynia caused by thalamic hemorrhage (10). Glial activation lasting for at least 3 months was also noted in adjacent areas following collagenase injection into the thalamic VPL of rhesus macaques, a primate model more directly comparable to humans (11). Microglia-mediated inflammatory cascades following stroke thus likely contribute to the genesis and maintenance of CPSP. However, the detailed mechanisms of how microglia participate in CPSP are still incompletely understood.

Fgr, a member of the Src family nonreceptor tyrosine kinases (12), plays an important role in pathological inflammatory responses (13, 14). Whether Fgr contributes to CPSP, and thalamic pain in particular, remains unknown. In the present study, we first established whether the expression of Fgr at mRNA and protein levels was altered in the thalamus in a well-characterized mouse model of hemorrhage-induced thalamic pain induced by microinjection of type IV collagenase (Coll IV) or autologous whole blood into unilateral ventral posterior medial nucleus (VPM) and VPL of murine thalami (15, 16). We then examined whether pharmacological inhibition or genetic knockdown of thalamic Fgr affected hemorrhage-induced pain hypersensitivities. Finally, we elucidated how Fgr participated in hemorrhage-induced thalamic pain responses.

## Results

### Thalamic Fgr is increased after local hemorrhage.

To demonstrate the role of Fgr in hemorrhage-induced thalamic pain, we first examined the expression of Fgr in the thalamus after microinjection of Coll IV into thalamic VPM and VPL in mice, a preclinical animal model that mimics hemorrhage-induced thalamic pain in clinic cases (16). Consistent with previous studies (16, 17), unilateral microinjection of Coll IV, but not saline, led to robust and long-lasting mechanical allodynia demonstrated by marked increases in paw withdrawal frequencies in response to 0.07 g and 0.4 g von Frey filaments (Supplemental Figure 1, A and B; supplemental material available online with this article; https://doi.org/10.1172/jci.insight.139987DS1) and heat hyperalgesia and cold hyperalgesia evidenced by significant decreases in paw withdrawal latencies to heat (Supplemental Figure 1C) and cold (Supplemental Figure 1D) stimuli, respectively, on the contralateral side. These pain hypersensitivities occurred 1 day after microinjection and lasted for at least 14 days (Supplemental Figure 1, A–D). As expected, there were no significant changes in basal paw withdrawal responses on the ipsilateral side (Supplemental Figure 1, A–D).

**Figure 1:**
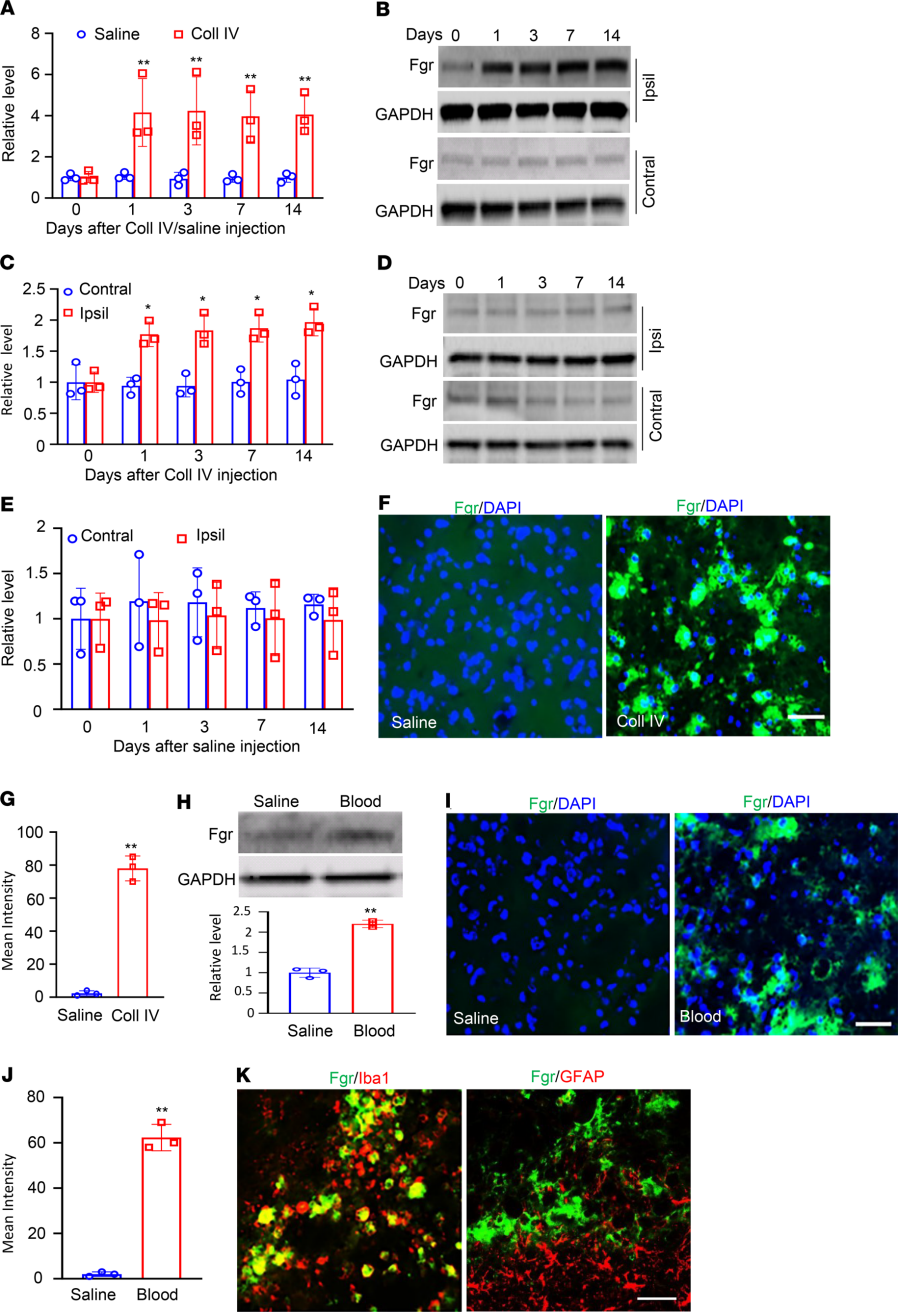
Thalamic Fgr was increased in both collagenase IV– and autologous blood–induced thalamic pain models. (**A**) Level of *Fgr* mRNA in the ipsilateral thalamus at different days after microinjection of Coll IV or saline. *n* = 3 biological repeats/group/time point. **P < 0.01 versus the corresponding control group (day 0) by 2-way ANOVA with repeated measures followed by post hoc Tukey’s test. (**B**–**E**) Amounts of Fgr protein in the ipsilateral (Ipsi) and contralateral (Contral) thalamus at different days after microinjection of Coll IV (**B** and **C**) or saline (**D** and **E**). Representative Western blots (**B** and **D**). Statistical summary of the densitometric analysis (**C** and **E**). *n* = 3 biological repeats/group/time point. *P < 0.05 versus the corresponding control group (day 0) by 2-way ANOVA with repeated measures followed by post hoc Tukey’s test. (**F** and **G**) Fgr immunofluorescence stainings of the ipsilateral thalamus on day 7 postmicroinjection of Coll IV or saline. Representative immunofluorescence stainings. Scale bar: 100 μm (**F**). Statistical summary of the densitometric analysis (**G**). *n* = 3 biological repeats/group. **P < 0.01 versus the saline group by 2-tailed unpaired Student’s *t* test. (**H**) The level of Fgr protein in the ipsilateral thalamus on day 3 postmicroinjection of autologous blood or saline. Representative Western blots (top). Statistical summary of the densitometric analysis (bottom). *n* = 3 biological repeats/group. **P < 0.01 versus the saline group by 2-tailed unpaired Student’s *t* test. (**I** and **J**) Fgr immunofluorescence stainings in the ipsilateral thalamus on day 3 postmicroinjection of autologous blood or saline. Representative immunoflourescence stainings. Scale bar: 100 μm (**I**). A statistical summary of the densitometric analysis (**J**). *n* = 3 biological repeats/group. **P < 0.01 versus the saline group by 2-tailed unpaired Student’s *t* test. (**K**) Fgr is colocalized with Iba1, but not GFAP, in the ipsilateral thalamus on day 3 after microinjection of Coll IV. Representative samples of *n* = 3 biological repeats. Scale bar: 100 μm.

Thalamic Fgr protein expression after Coll IV microinjection was then examined. Unilateral microinjection of Coll IV persistently increased the expression of *Fgr* mRNA and its coded Fgr protein in the thalamus on the ipsilateral, but not the contralateral, side (Figure 1, A–E). The levels of *Fgr* mRNA in the ipsilateral thalamus were increased by 3.8-, 4.5-, 4.2-, and 4.1-fold on days 1, 3, 7, and 14 after Coll IV microinjection, respectively, compared with those after saline microinjection at the corresponding time points (Figure 1A). The amount of Fgr protein in the ipsilateral thalamus was increased by 1.9-, 2.0-, 1.9-, and 2.0-fold on days 1, 3, 7, and 14 after Coll IV microinjection, respectively, as compared with the corresponding time points in the contralateral thalamus (Figure 1, B and C). As expected, saline microinjection did not alter basal levels of *Fgr* mRNA or Fgr protein in the thalamus on either side during the observation period (Figure 1, A, D, and E). Consistently, Fgr-labeled immunoreactivities were very weak or undetectable in naive mice or the saline-microinjected group (Figure 1F). Coll IV microinjection robustly increased the density of Fgr-labeled immunoreactivities in the ipsilateral thalamus on day 7 by 35-fold as compared with the corresponding saline-microinjected group (Figure 1, F and G). These increases occurred in the regions around/adjacent to the core of hemorrhagic lesions in thalamic VPM and VPL (Supplemental Figure 2). Results were similar after microinjection of autologous whole blood into thalamic VPM and VPL (Figure 1, H–J), another preclinical animal model of hemorrhage (15, 16). The levels of Fgr protein and the density of Fgr-labeled immunoreactivities in the ipsilateral thalamus were increased by 2.2-fold (Figure 1H) and 31-fold (Figure 1, I and J), respectively, on day 3 after whole blood microinjection as compared with the saline microinjection. A double labeling assay showed that the majority of Fgr-labeled immunoreactivities overlapped with immunoreactivities labeled by ionized calcium-binding adapter molecule 1 (Iba1) (a marker for microglia) and few with immunoreactivities labeled by glial fibrillary acidic protein (GFAP) (a marker for astrocytes), CD68 (a marker for microphages), or NeuN (a marker for neurons) in thalamic cells 7 days after Coll IV microinjection (Figure 1K and Supplemental Figure 3). This finding indicates that increased Fgr is expressed predominantly in thalamic microglia.

### Pharmacological inhibition of Fgr attenuates hemorrhage-induced thalamic pain genesis and maintenance.

**Figure 2:**
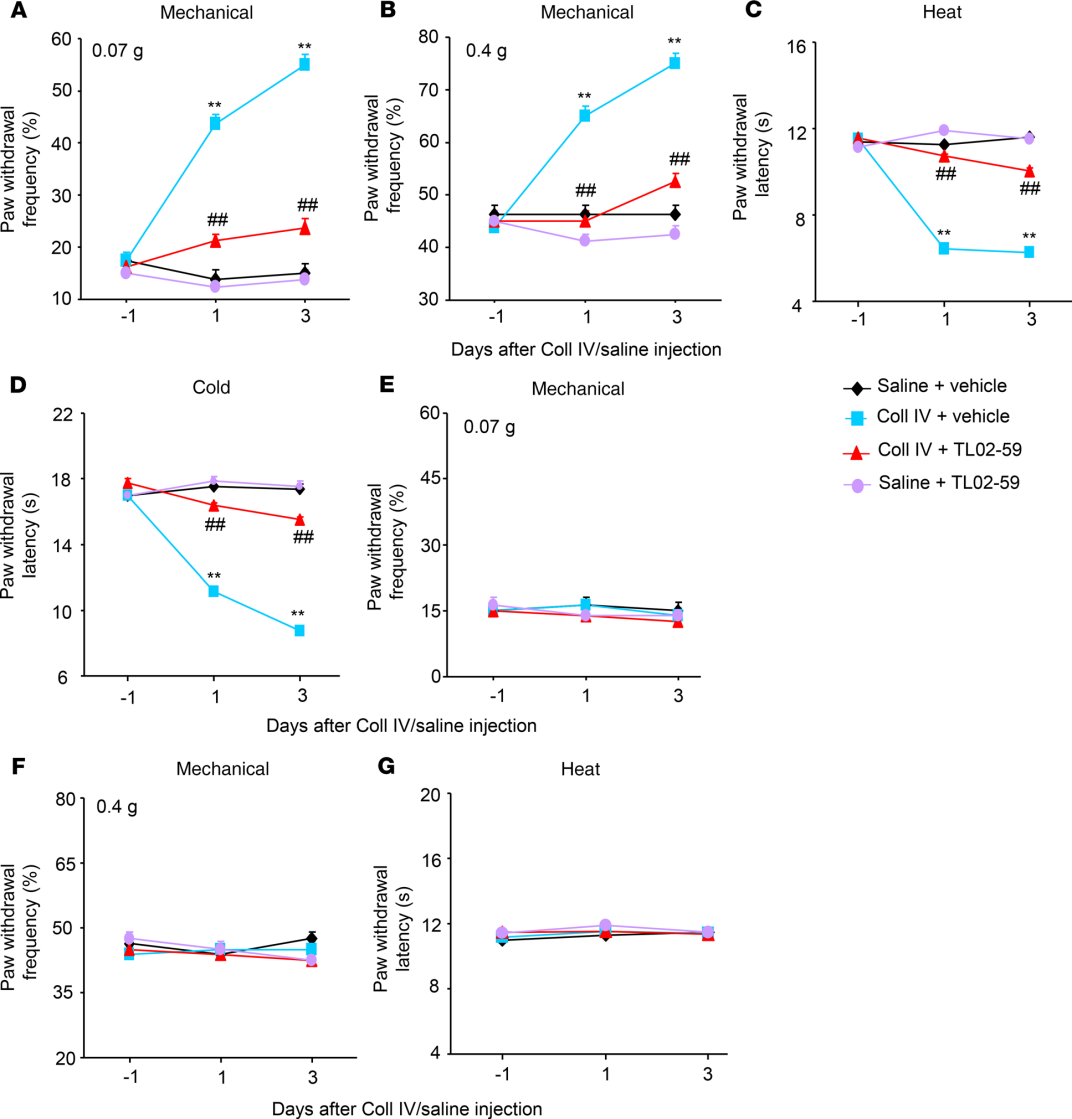
Effect of systemic tail vein administration of TL02-59 on Coll IV microinjection–induced thalamic pain development. TL02-59 (15 mg/kg) or vehicle was given 30 minutes before Coll IV or saline microinjection and once daily post–Coll IV or saline microinjection. Effect of systemic preadministration of TL02-59 or vehicle on paw withdrawal frequencies to 0.07 g (**A** and **E**) and 0.4 g (**B** and **F**) von Frey filaments and paw withdrawal latencies to heat (**C** and **G**) and cold (**D**) stimuli on days 1 and 3 after thalamic microinjection of Coll IV or saline on the contralateral (**A**–**D**) and ipsilateral (**E**–**G**) sides. *n* = 8 mice/group. Two-way ANOVA with repeated measures followed by post hoc Tukey’s test. **P < 0.01 versus the corresponding baseline (day –1). ^##^*P* < 0.01 versus the Coll IV plus vehicle group at the corresponding days.

**Figure 3:**
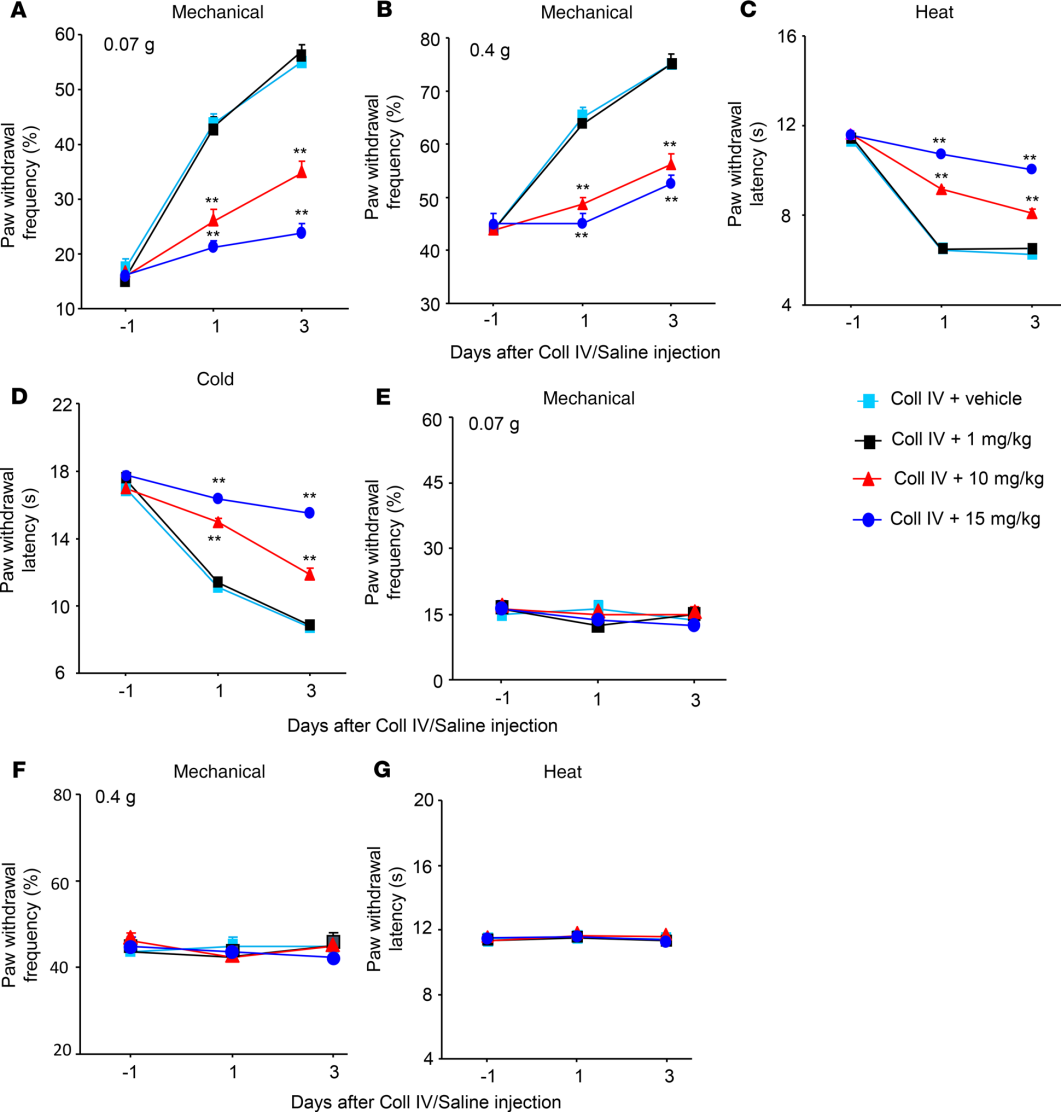
Dose-dependent effect of systemic tail vein administration of TL02-59 on Coll IV microinjection–induced thalamic pain development. Effect of systemic preadministration of TL02-59 (0 [vehicle], 1, 10, and 15 mg/kg) on paw withdrawal frequencies to 0.07 g (**A** and **E**) and 0.4 g (**B** and **F**) von Frey filaments and paw withdrawal latencies to heat (**C** and **G**) and cold (**D**) stimuli on days 1 and 3 after thalamic microinjection of Coll IV on the contralateral (**A**–**D**) and ipsilateral (**E**–**G**) sides. *n* = 8 mice/group. Two-way ANOVA with repeated measures followed by post hoc Tukey’s test. **P < 0.01 versus the Coll IV plus vehicle group at the corresponding days.

We further examined whether the increased thalamic Fgr participated in hemorrhage-induced thalamic pain genesis. TL02-59 (1, 10, or 15 mg/kg; dissolved in 90% saline, 5% solutol HS-15 and 5% *N*-methyl-2-pyrrolidone), a selective inhibitor of Fgr (18), as well as vehicle (5% solutol HS-15 and 5% *N*-methyl-2-pyrrolidone), were administered through the tail vein 30 minutes before microinjection of Coll IV or saline and once daily for 3 days thereafter. Behavioral tests were carried out 1 day before Coll IV/saline microinjection and 30 minutes post–TL02-59/vehicle administration on days 1 and 3 after Coll IV/saline microinjection. As expected, mechanical allodynia, heat hyperalgesia, and cold hyperalgesia were seen on the contralateral (but not ipsilateral) side of the vehicle plus Coll IV–treated group on days 1 and 3 post–Coll IV microinjection (Figure 2, A–G). These pain hypersensitivities were significantly attenuated in the Coll IV–microinjected mice with systemic administration of TL02-59 at 15 mg/kg (Figure 2, A–D). These effects were dose dependent (Figure 3, A–D). TL02-59 at these doses did not alter basal paw withdrawal frequencies and latencies on the ipsilateral side of the TL02-59 plus Coll IV–treated group (Figure 2, E–G; Figure 3, E–G) and on both contralateral (Figure 2, A–D) and ipsilateral (Figure 2, E–G) sides of the TL02-59 plus saline–treated group during the observation period. As expected, vehicle administration did not affect basal paw withdrawal responses on either side in the saline-treated mice (Figure 2, A–G). The treated mice displayed normal locomotor activity (Table 1).

**Figure 4:**
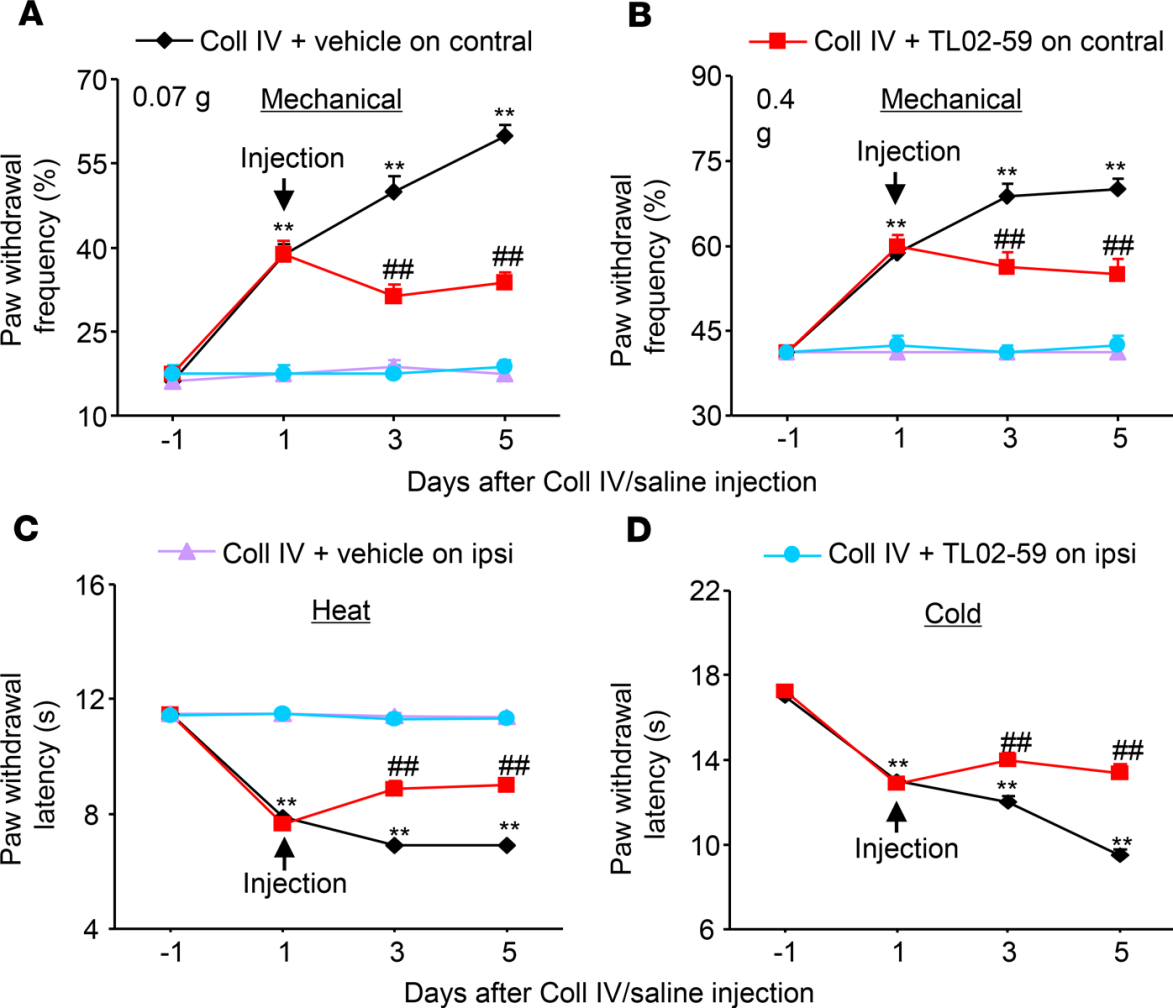
Effect of systemic tail vein administration of TL02-59 on Coll IV microinjection–induced thalamic pain maintenance. TL02-59 (15 mg/kg) or vehicle was given 1 day after Coll IV microinjection and once daily post–Coll IV microinjection for 5 days. Effect of systemic preadministration of TL02-59 or vehicle on paw withdrawal frequencies to 0.07 g (**A**) and 0.4 g (**B**) von Frey filaments and paw withdrawal latencies to heat (**C**) and cold (**D**) stimuli on days 3 and 5 after thalamic microinjection of Coll IV on the contralateral and ipsilateral sides. *n* = 8 mice/group. Two-way ANOVA with repeated measures followed by post hoc Tukey’s test. **P < 0.01 versus the corresponding baseline (day –1). ^##^*P* < 0.01 versus the Coll IV plus vehicle group at the corresponding days.

The role of thalamic Fgr in hemorrhage-induced thalamic pain maintenance was observed. TL02-59 (15 mg/kg) and vehicle were administered through the tail vein 1 day after microinjection of Coll IV and once daily for 5 days. At 1 day post–Coll IV microinjection, mechanical allodynia, heat hyperalgesia, and cold hyperalgesia were robustly developed (Figure 2, Figure 3, and Figure 4). Systemic postadministration of TL02-59 at the dose used significantly blocked these pain hypersensitivities on days 3 and 5 post–Coll IV microinjection on the contralateral side (Figure 4, A–D). As expected, mechanical allodynia, heat hyperalgesia, and cold hyperalgesia were still seen on days 3 and 5 post–Coll IV microinjection in the vehicle-treated group (Figure 4, A–D). Neither vehicle nor TL02-59 altered basal paw withdrawal responses on the ipsilateral side (Figure 4, A–D).

**Figure 5:**
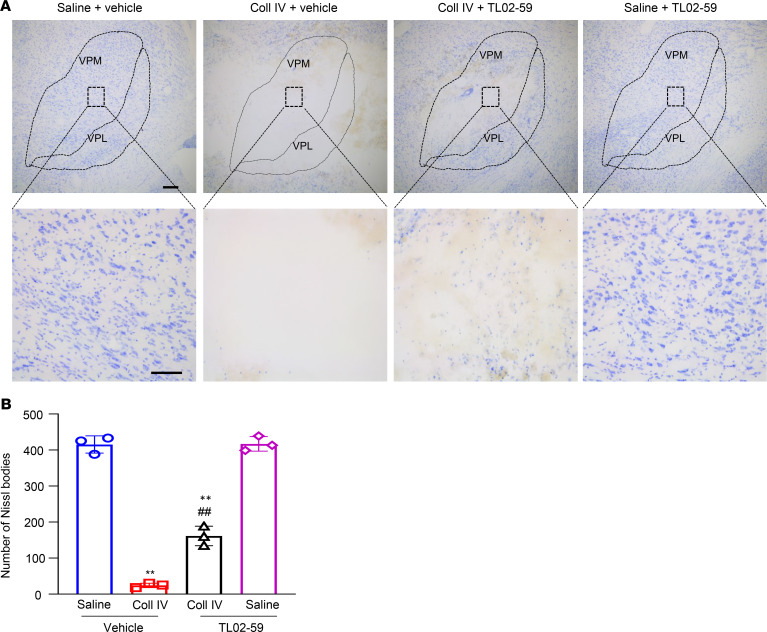
Effect of systemic tail vein administration of TL02-59 (15 mg/kg) on Coll IV microinjection–induced thalamic lesion. (**A**) Representative coronal brain sections stained with Nissl from the different treatment groups on day 3 after thalamic microinjection of Coll IV or saline. Top: thalamic sections including VPL and VPM. Scale bar: 100 μm. Bottom: magnification of the corresponding top photographs. Scale bar: 50 μm. (**B**) Number of Nissl-stained cells in the VPL and VPM of thalami from the different treatment groups as indicated. *n* = 3 biological repeats/group. Two-way ANOVA with repeated measures followed by post hoc Tukey’s test. **P < 0.01 versus the saline plus vehicle–treated group. ^##^*P* < 0.01 versus the Coll IV plus vehicle–treated group.

We also examined whether systemic administration of TL02-59 improved Coll IV microinjection–induced thalamic injury. After behavioral tests, brain tissues were collected for Nissl staining. As shown in Figure 5, A and B, numerous Nissl-stained cells were observed in the ipsilateral VPL and VPM of the saline plus vehicle– or TL02-59–treated groups. Few Nissl-stained cells were seen in the ipsilateral VPL and VPM of the Coll IV plus vehicle–treated group (Figure 5, A and B). Compared with the Coll IV plus vehicle–treated group, the number of Nissl-stained cells detected was significantly increased in the ipsilateral VPL and VPM of the Coll IV plus TL02-59 treatment group, although it was still markedly lower than that in the saline-treated groups (Figure 5, A and B). Most of these Nissl-stained cells were positive for Iba1, with a few positive for GFAP and CD68 and none for NeuN (Supplemental Figure 4). In addition, there were visible differences in the areas of tissue damages between the Coll IV plus vehicle–treated group and the Coll IV plus TL02-59–treated group (Figure 5A). As expected, there were no profound changes in number of Nissl-stained cells in the contralateral VPL and VPM among the treated groups (Supplemental Figure 5, A and B).

**Figure 6:**
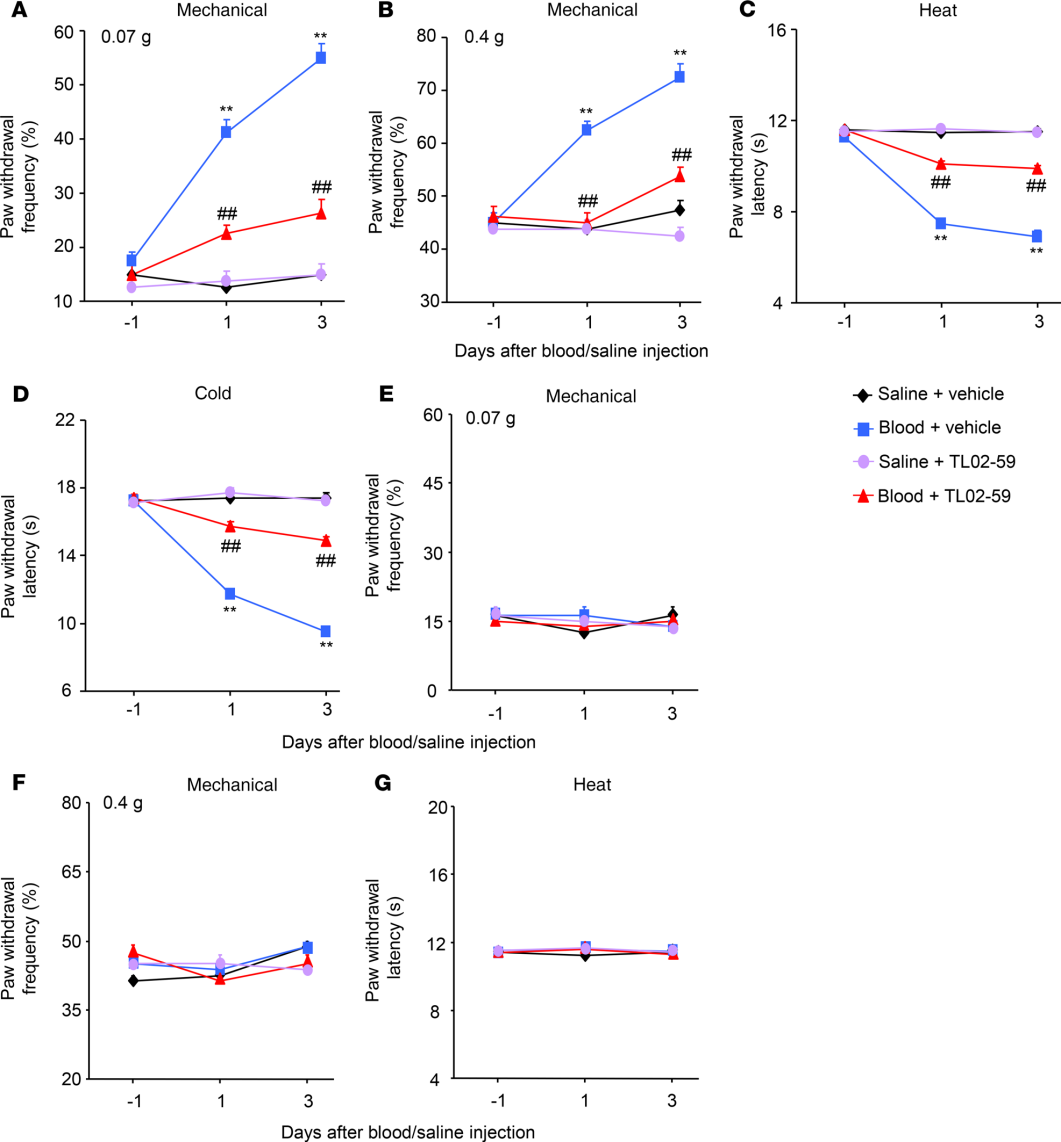
Effect of systemic tail vein administration of TL02-59 on autologous blood microinjection–induced thalamic pain genesis. TL02-59 or vehicle was given 30 minutes before autologous blood or saline microinjection and once daily thereafter. Effect of systemic administration of TL02-59 (15 mg/kg) or vehicle on paw withdrawal frequencies to 0.07 g (**A** and **E**) and 0.4 g (**B** and **F**) von Frey filaments and paw withdrawal latencies to heat (**C** and **G**) and cold (**D**) stimuli on days 1 and 3 after thalamic microinjection of autologous blood or saline on the contralateral (**A**–**D**) and ipsilateral (**E**–**G**) sides. *n* = 8 mice/group. Two-way ANOVA with repeated measures followed by post hoc Tukey’s test. **P < 0.01 versus the corresponding baseline (day –1). ^##^*P* < 0.01 versus the autologous blood plus vehicle group at the corresponding days.

Similar behavioral response and thalamic injury attenuation were seen after systemic administration of TL02-59 via tail vein injection in the autologous whole-blood microinjection–induced thalamic pain model (Figure 6, A–G; Supplemental Figure 6, A and B; Supplemental Figure 7, A and B; Table 1).

### Knocking down thalamic Fgr attenuates the hemorrhage-induced thalamic pain genesis.

**Figure 7:**
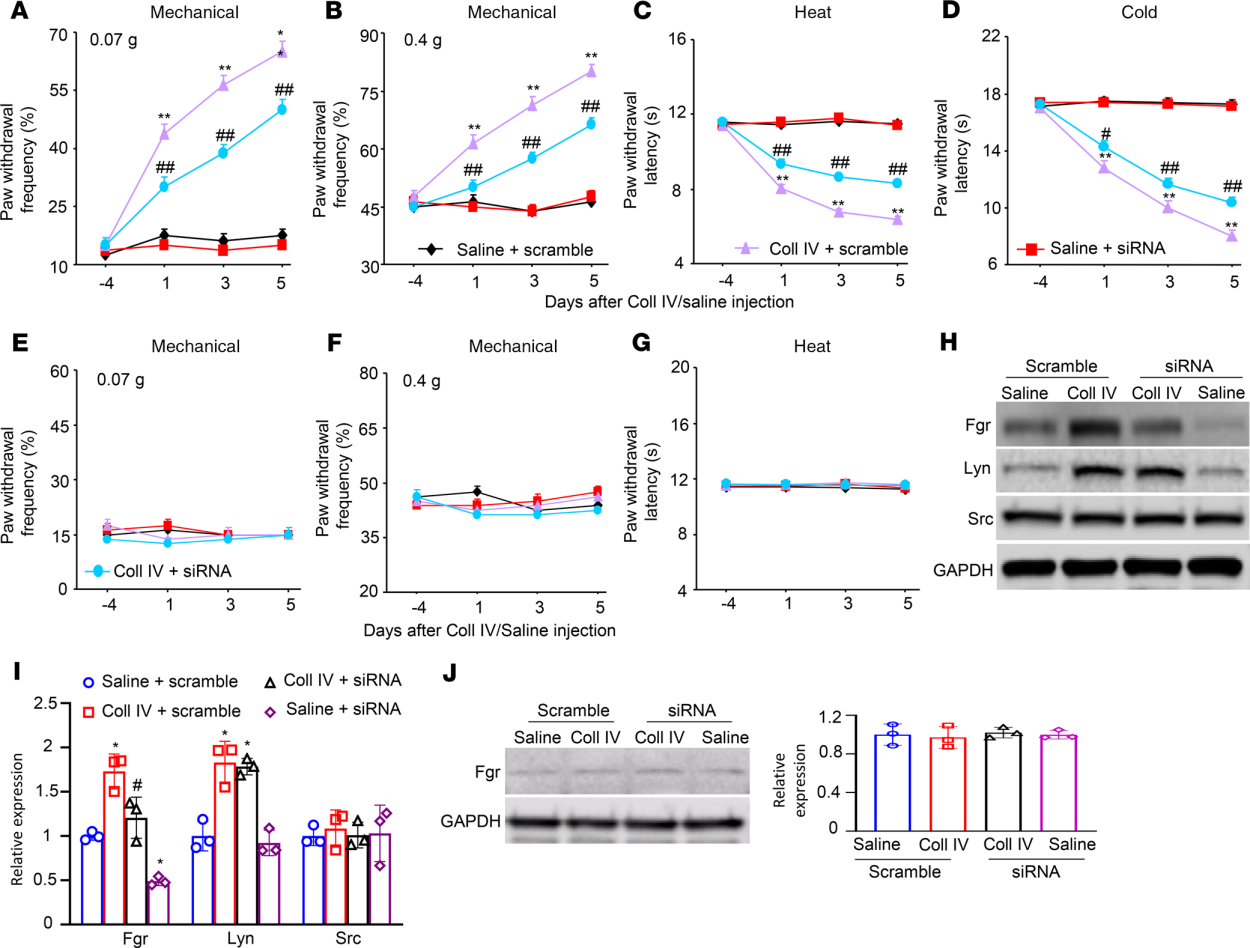
Effect of thalamic microinjection of *Fgr* siRNA on Coll IV microinjection–induced thalamic pain genesis. (**A**–**G**) Effect of microinjection of *Fgr* siRNA (160 μM, 0.5 μL) or control scrambled siRNA (160 μM, 0.5 μL) into the VPL and VPM of the unilateral thalamus 4 days before Coll IV or saline microinjection into the same regions on paw withdrawal frequencies to 0.07 g (**A** and **E**) and 0.4 g (**B** and **F**) von Frey filaments and paw withdrawal latencies to heat (**C** and **G**) and cold (**D**) stimuli on the contralateral (**A**–**D**) and ipsilateral (**E**–**G**) sides on days 1, 3, and 5 after Coll IV/saline microinjection. *n* = 8 mice/group. Two-way ANOVA with repeated measures followed by post hoc Tukey’s test. **P < 0.01 versus the corresponding baseline (day –4). ^##^*P* < 0.01 versus the Coll IV plus vehicle group at the corresponding days. (**H**–**J**) Effect of microinjection of *Fgr* siRNA (160 μM, 0.5 μL) or control scrambled siRNA (160 μM, 0.5 μL) into unilateral thalamic VPL and VPM 4 days before Coll IV or saline microinjection into the same regions on the expression of Fgr, Lyn, and Src proteins in the ipsilateral (**H** and **I**) and contralateral (**J**) thalamus on day 5 post–Coll IV or saline microinjection. *n* = 3 biological repeats/group. Two-way ANOVA with repeated measures followed by post hoc Tukey’s test. *P < 0.05 versus the corresponding saline plus scrambled siRNA-treated group. ^#^*P* < 0.05 versus the corresponding Coll IV plus scrambled siRNA-treated group.

An important limitation of systemic administration of TL02-59 is the lack of anatomical and pharmacological specificity. To further confirm the role of thalamic Fgr in hemorrhage-induced thalamic pain, we knocked down thalamic Fgr through microinjection of *Fgr* siRNA into the VPL and VPM of unilateral thalamus 4 days before ipsilateral microinjection of Coll IV or saline into the same regions. *Fgr* scrambled siRNA was used as a negative control. Consistent with the observations above, Coll IV microinjection produced significant increases in paw withdrawal frequencies to 0.07 g and 0.4 g von Frey filament stimuli and reductions in paw withdrawal latencies to heat and cold stimuli at days 1, 3, and 5 post–Coll IV microinjection on the contralateral side in *Fgr* scrambled siRNA–pretreated mice (Figure 7, A–D). However, the Coll IV mice premicroinjected with *Fgr* siRNA exhibited attenuation of the contralaterally increased paw withdrawal responses to 0.07 g and 0.4 g von Frey filament stimuli and of the contralaterally reduced paw withdrawal latencies to heat and cold stimuli at days 1 to 5 post–Coll IV microinjection as compared with the *Fgr* scrambled siRNA–microinjected Coll IV mice at the corresponding time points (Figure 7, A–D). Neither siRNA affected locomotor function (Table 1) and basal paw withdrawal responses to mechanical, heat, and cold stimuli on the ipsilateral side of Coll IV mice (Figure 7, E–G) or on the contralateral and ipsilateral sides of saline-injected controls (Figure 7, A–G) during the observation period.

After behavior testing we harvested the ipsilateral thalami on day 5 post–Coll IV/saline microinjection and first assessed the expression of Fgr in the thalamus. As expected, the amount of Fgr protein was increased by 1.7-fold in the ipsilateral thalamus of the *Fgr* scrambled siRNA–treated Coll IV mice as compared with the *Fgr* scrambled siRNA–treated saline mice (Figure 7, H and I). This increase was not seen in the Coll IV–treated mice premicroinjected with *Fgr* siRNA (Figure 7, H and I). The level of Fgr protein in the ipsilateral thalamus of the *Fgr* siRNA–microinjected Coll IV mice was decreased by 29% compared with the *Fgr* scrambled siRNA–microinjected Coll IV mice. Lyn and Src are another 2 members of the Src family nonreceptor tyrosine kinases (12). Although Coll IV microinjection also increased the expression of Lyn (but not Src) in the thalamus of the *Fgr* scrambled siRNA–treated mice (Figure 7, H and I), thalamic premicroinjection of *Fgr* siRNA did not affect the expression of the increased Lyn and basal Src in the ipsilateral thalamus of the Coll IV mice (Figure 7, H and I). In addition, thalamic microinjection of neither siRNA altered basal expression of Fgr in the contralateral thalamus (Figure 7J).

We also carried out the Nissl staining after behavioral tests and examined whether *Fgr* siRNA microinjection improved thalamic Coll IV–induced hemorrhagic injury. As expected, few Nissl-stained cells were observed in the ipsilateral thalamic VPL and VPM of the *Fgr* scrambled siRNA–treated Coll IV mice, whereas numerous Nissl-stained cells were seen in the same regions of the *Fgr* scrambled siRNA– or Fgr siRNA–treated saline mice (Supplemental Figure 8, A and B). Compared with the *Fgr* scrambled siRNA–treated Coll IV mice, the number of Nissl-stained cells was markedly increased in the ipsilateral thalamic VPL and VPM of the Coll IV mice premicroinjected with *Fgr* siRNA, although it was still significantly lower than that in the *Fgr* scrambled siRNA–treated saline mice (Supplemental Figure 8, A and B). Interestingly, there were no visible differences in the areas of tissue damage between the scrambled siRNA–treated and *Fgr* siRNA–treated Coll IV mice (Supplemental Figure 8A). As expected, neither siRNA altered number of Nissl-stained cells in the contralateral VPL and VPM among the treated groups (Supplemental Figure 9, A and B).

### Hemorrhage-induced increase in Fgr activates NF-κB and ERK1/2 pathways in thalamic microglia.

**Figure 8:**
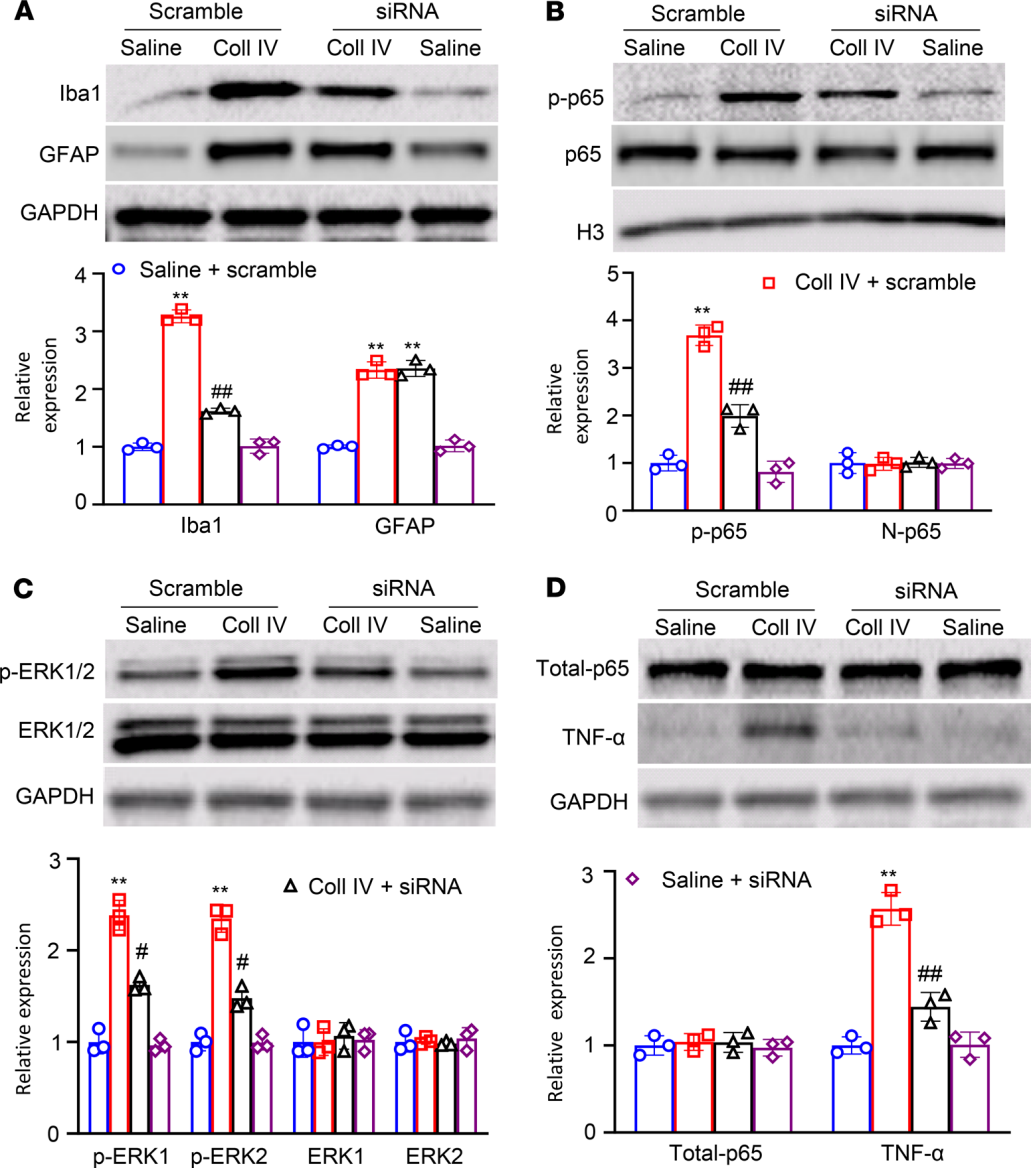
Effect of thalamic microinjection of *Fgr* siRNA on Coll IV microinjection–induced thalamic activation of NF-κB and ERK1/2 pathways and production of TNF-α. Expression of Iba1 and GFAP in the cytoplasmic fraction (**A**), expression of phosphorylated p65 (p-p65) and p-65 in the nuclear fraction (**B**), expression of phosphorylated ERK1/2 (p-ERK1/2) and ERK1/2 in the cytoplasmic fraction (**C**), and expression of p65 (total-p65) and TNF-α in the total cellular fraction (**D**) from the ipsilateral thalamus of the treatment groups on day 5 after thalamic microinjection of collagenase type IV (Coll IV) or saline. Top: representative Western blots. Bottom: statistical summary of the densitometric analysis. *n* = 3 biological repeats/group. Two-way ANOVA with repeated measures followed by post hoc Tukey’s test. **P < 0.01 versus the corresponding saline plus scrambled siRNA-treated group. ^#^*P* < 0.05, ^##^*P* < 0.01 versus the corresponding Coll IV plus scrambled siRNA-treated group.

**Figure 9:**
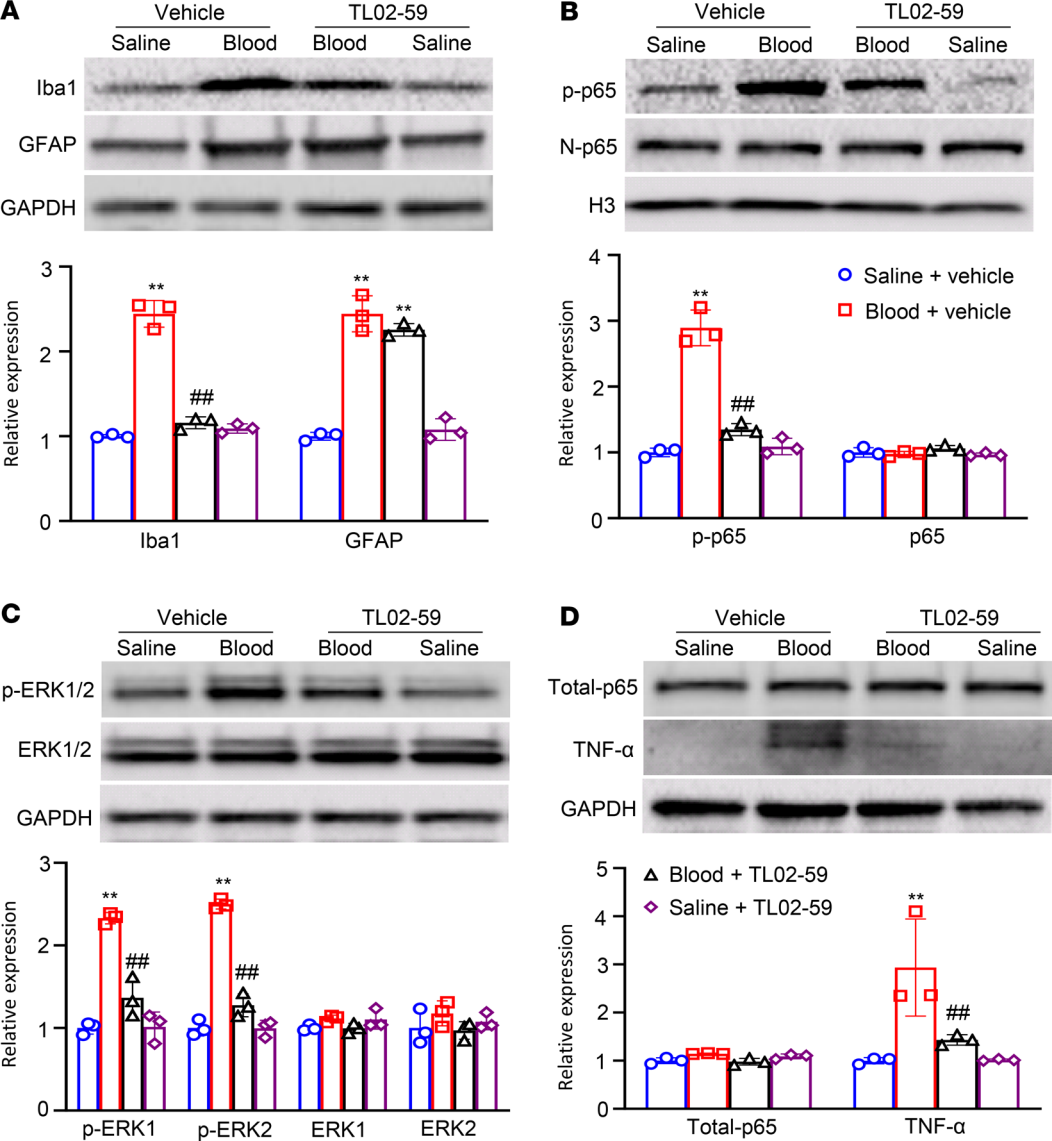
Effect of systemic tail vein administration of TL02-59 on autologous blood microinjection–induced thalamic activation of NF-κB and ERK1/2 pathways and production of TNF-α. Expression of Iba1 and GFAP in the cytoplasmic fraction (**A**), expression of p-p65 and p-65 in the nuclear fraction (**B**), expression of p-ERK1/2 and ERK1/2 in the cytoplasmic fraction (**C**) and expression of p65 (total-p65) and TNF-α in the total cellular fraction (**D**) from the ipsilateral thalamus of the different treatment groups on day 3 after thalamic microinjection of autologous blood or saline. Top: representative Western blots. Bottom: statistical summary of the densitometric analysis. *n* = 3 biological repeats/group. Two-way ANOVA with repeated measures followed by post hoc Tukey’s test. **P < 0.01 versus the corresponding saline plus vehicle–treated group. ^##^*P* < 0.01 versus the corresponding autologous blood plus vehicle–treated group.

**Figure 10:**
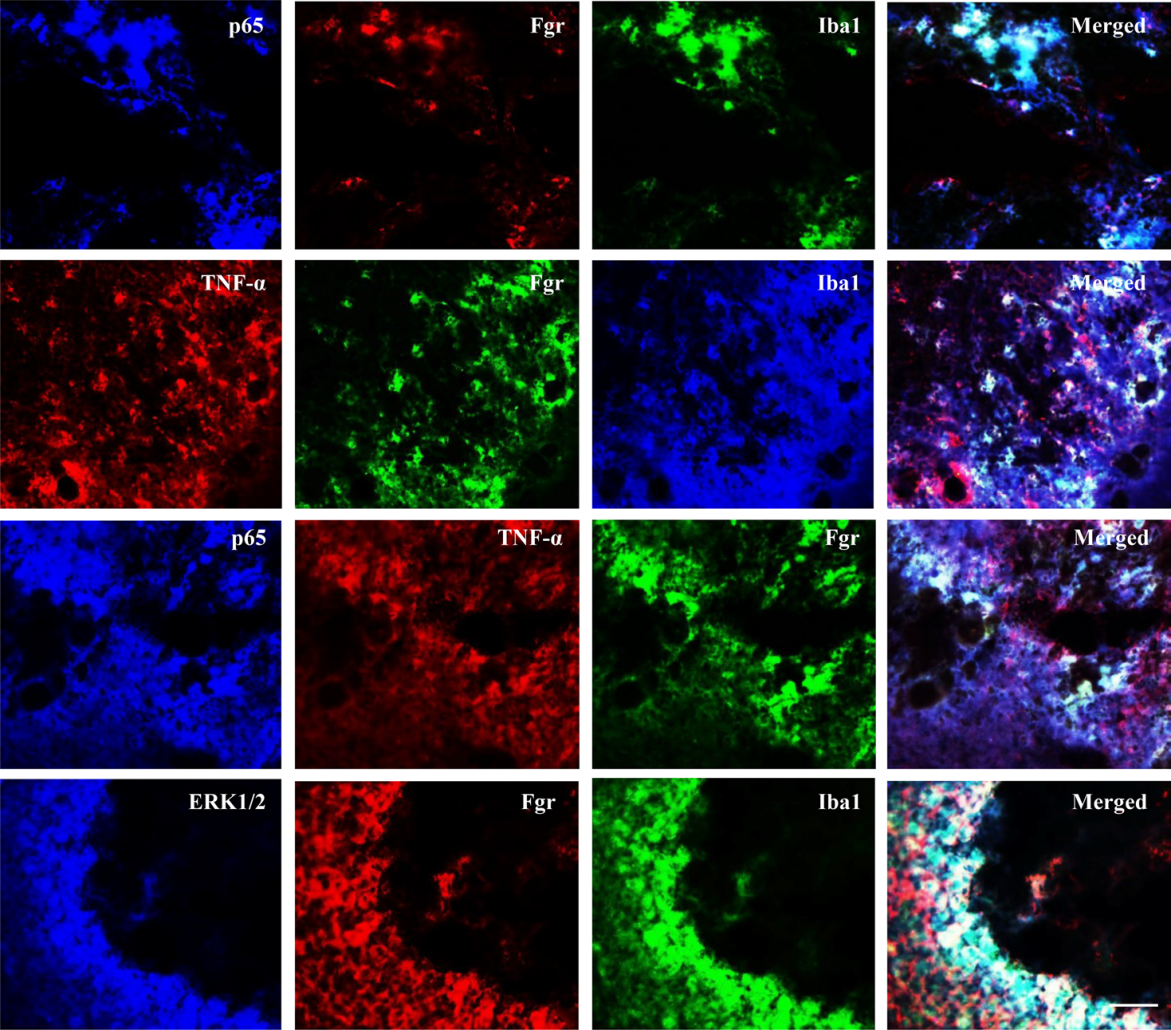
Coexpression of Fgr with p65, TNF-α, or ERK1/2 and coexpression of p65 with TNF-α in microglia of hemorrhagic thalami. Triple immunofluorescence staining showing coexpression of Iba1 with p-65 and Fgr, with TNF-α and Fgr, and with ERK1/2 and Fgr as well as of Fgr with p65 and TNF-α in the regions adjacent to the core of thalamic hemorrhagic injury on day 5 post–Coll IV microinjection. Representative images from 3 biological repeats (*n* = 3 mice). Scale bar: 100 μm.

Finally, we explored how the increased Fgr contributed to hemorrhage-induced thalamic pain genesis. Consistent with previous observations (9, 10, 19), Coll IV microinjection led to the activation of microglia and astrocytes as evidenced by significant increases in the levels of Iba1 and GFAP, respectively, in the ipsilateral thalami of *Fgr* scrambled siRNA–treated mice on day 5 post–Coll IV microinjection (Figure 8A). The increased expression of Iba1, but not of GFAP, was attenuated by thalamic premicroinjection of *Fgr* siRNA in the Coll IV–treated mice (Figure 8A). Moreover, Coll IV microinjection activated NF-κB and ERK pathways as documented by the increased amounts of phosphorylated p65 (p-p65), a key member of NF-κB family (20, 21), in the nuclear fraction and of phosphorylated ERK1/2 (p-ERK1/2) in the total cellular fraction, respectively, in the ipsilateral thalami of *Fgr* scrambled siRNA–treated mice on day 5 post–Coll IV microinjection (Figure 8, B and C). These increases were significantly inhibited by thalamic premicroinjection of *Fgr* siRNA in the Coll IV–treated mice (Figure 8, B and C). As expected, Coll IV microinjection did not alter basal expression of p65 in the nuclear fraction and basal expression of p65 and ERK1/2 in the total cellular fraction from the ipsilateral thalami of the *Fgr* scrambled siRNA–treated mice (Figure 8, B–D). Interestingly, the level of TNF-α in the total cellular fraction from the ipsilateral thalamus of *Fgr* scrambled siRNA mice on day 5 post–Coll IV microinjection was increased by 2.6-fold as compared with the corresponding *Fgr* scrambled siRNA–treated saline mice (Figure 8, B–D). Thalamic premicroinjection of *Fgr* siRNA almost entirely abolished this increase on day 5 post–Coll IV microinjection (Figure 8D). Similar expression patterns of Iba1, GFAP, p-p65, p65, p-ERK1/2, ERK1/2, and TNF-α in the ipsilateral thalami were seen after systemic preadministration of TL02-59 via tail vein injection in the autologous whole blood microinjection–induced thalamic pain model (Figure 9, A–D). Triple immunohistochemical staining showed that Iba1-labeled immunoreactivities overlapped with immunoreactivities labeled by p65 and Fgr, TNF-α and Fgr, or ERK1/2 and Fgr in the regions adjacent to the core of hemorrhagic lesions in the thalamus on day 5 post–Coll IV microinjection (Figure 10). In addition, Fgr-labeled immunoreactivities overlapped with immunoreactivities labeled by p65 and TNF-α in this region (Figure 10).

## Discussion

Microinjection of either Coll IV or autologous whole blood into unilateral VPL and VPM regions of murine thalami leads to long-lasting pain hypersensitivities, including mechanical allodynia, heat hyperalgesia, and cold hyperalgesia on the contralateral side, which mimics thalamic pain caused by a hemorrhagic stroke in humans (15–17, 22). Understanding the mechanisms of how this pain hypersensitivity genesis occurs may allow the development of novel therapeutic treatments for thalamic pain. Although several studies on hemorrhage-induced thalamic pain have been carried out during the past decades (23, 24), the detailed mechanisms remain elusive. In this study, we found that thalamic microinjection of Coll IV or autologous whole blood produced an increase in Fgr expression at both mRNA and protein levels in the ipsilateral thalamus and that this increase contributed to Coll IV– or autologous whole blood–induced induction and maintenance of pain hypersensitivity likely through the NF-κB- and ERK1/2-triggered production of TNF-α in thalamic microglia. Our findings suggest that Fgr is a key player in hemorrhage-induced thalamic pain.

Fgr is 1 of at least 9 members of the Src-family nonreceptor tyrosine kinases (SFKs). At least 6 SFK members, Src, Fyn, Lck, Yes, Yrk, and Lyn, are ubiquitously expressed in the central nervous system, whereas Fgr, Blk, and Hck are only expressed in specific tissues (25). These kinases participate in regulating cell growth, proliferation, metabolism, differentiation, and migration (25, 26). Src and Fyn have also been implicated in neuronal development and synaptic plasticity (27, 28). More importantly, Src, Fyn, and Lyn are each essential for the development of chronic pain caused by complete Freund’s adjuvant injection, peripheral nerve injury, streptozotocin injection, and bone metastasis (27–29). However, whether and how Fgr contributes to chronic pain have not been reported previously to our knowledge.

The *Fgr* gene in the thalamus can be activated at the transcriptional level in response to hemorrhagic stroke. The present study showed that the level of Fgr expression was rather low in naive thalami, as Fgr immunostaining was very weak or undetectable in the thalamus under normal conditions. However, after Coll IV or autologous blood was microinjected into the thalamus, the amounts of *Fgr* mRNA and Fgr protein were markedly and persistently increased in the ipsilateral, but not the contralateral, thalamic nuclei. By double-labeled immunostaining, this increase was demonstrated to occur predominantly in thalamic microglia. How the *Fgr* gene is activated following hemorrhagic stroke is still unknown. Potential mechanisms, including changes in epigenetic modifications and increases in transcription factor expression and/or mRNA stability that may lead to an elevation of *Fgr* mRNA following a hemorrhagic stroke, cannot be ruled out and remain open to further investigation.

The contribution of the increased thalamic Fgr to Coll IV– or autologous blood–induced pain hypersensitivity was demonstrated in the present study, not only by pharmacological Fgr inhibition through systemic tail vein administration of the specific Fgr inhibitor TL02-59 but also by blocking the increased Fgr through microinjection of *Fgr* siRNA into the ipsilateral thalamus. Pharmacological effects were dose dependent. Because the hemorrhage causes dysfunction or disruption of the blood-brain barrier (30–32), systemic administration of TL02-59 likely acts on thalamic microglia directly. Nerveless, its effect on circulating immune cells (e.g., monocytes) could not be excluded. Consistently, the damage to thalamic cells caused by microinjection of Coll IV or autologous blood was also reduced, as many Nissl-stained cells were detected in the VPL and VPM, after pretreatment with systemic TL02-59 or thalamic premicroinjection of *Fgr* siRNA. We further identified that most of these Nissl-stained cells were microglia, with a few astrocytes and microphages and no neurons. These data suggest that Fgr inhibition or knockdown predominantly protects microglia from the Coll IV–/autologous blood–induced glial damage in the VPL and VPM. This selective protection may be related to main expression of Fgr in thalamic microglia. We found that *Fgr* siRNA’s effects were less strong than systemic TL02-59’s effects. This may be due to microinjection of a small limited volume (0.5 μL) of siRNA into thalamus, resulting in partial thalamic microglia being targeted. It should be noted that both systemic administration of TL02-59 and thalamic microinjection of *Fgr* siRNA profoundly attenuated, not abolished, Coll IV– or autologous blood–induced pain hypersensitivities and thalamic cell damage. These partial effects may be related to multiple mechanisms contributing to the development of hemorrhagic stroke–induced thalamic pain. The present study also revealed that the thalamic expression of Lyn, but not Src, was increased after thalamic microinjection of Coll IV. This kinase thus likely participates in thalamic pain development and maintenance, but this needs to be verified in future research. In addition, increased synaptic glutamate release and subsequently excessive activation of excitatory postsynaptic glutamate receptors (e.g., NMDA receptors) and extreme Ca^2+^ influx into thalamic cells through these receptors following thalamic hemorrhage have been proposed to exacerbate the infarction and be involved in thalamic pain genesis (16, 33). Therefore, combined strategies that target different mechanisms underlying thalamic pain may produce great benefits in the management of this disorder.

The production of TNF-α triggered via the activations of both NF-κB and ERK1/2 pathways in thalamic microglia may mediate the role of the increased Fgr in hemorrhage-induced thalamic pain. Prolonged microglial activation is implicated in thalamic pain development because microglial inhibition or depletion ameliorates pain hypersensitivities caused by thalamic microinjection of Coll IV (9, 10). The chemokines and cytokines released from microglia are involved in hemorrhage-induced thalamic pain (34). However, how thalamic microglia are activated and triggered to release these chemokines and cytokines following hemorrhagic stroke is unclear. We revealed that pharmacological inhibition or genetic knockdown of the increased Fgr blocked the Coll IV– and autologous blood–induced increases in the level of Iba1, but not GFAP, in the thalamus. This indicates that activation of microglia, but not astrocytes, may be dependent on increased thalamic Fgr. The present study also revealed that blocking thalamic Fgr attenuated the Coll IV– and autologous blood–induced increase in thalamic TNF-α. TNF-α, a proinflammatory cytokine, may be induced via activation of NF-κB and ERK1/2 pathways following exposure to immune stimulants including bacterial endotoxin lipopolysaccharide (35) and is a trigger of peripheral neuropathic pain (36). NF-κB, a nuclear transcription factor, controls numerous genes encoding inflammatory cytokines and nociceptive mediators and plays a key role in the genesis of peripheral neuropathic pain (20, 37). ERK1/2 also activates several transcription factors, regulates gene transcription and translation, and participates in the production of cytokines under peripheral neuropathic pain conditions (38, 39). Given that Fgr was coexpressed with p65, ERK1/2, and TNF-α, and that p65 was also coexpressed with TNF-α in thalamic microglia under hemorrhagic stroke conditions, Fgr likely participates in the hemorrhage-induced microglial activation and subsequent production of TNF-α via the activation of both NF-κB and ERK1/2 pathways. Indeed, the present study demonstrated that thalamic microinjection of Coll IV or autologous blood increased the levels of p–NF-κB subunit p-p65 (activated form) in the nucleus and the amount of p-ERK1/2 (activated form) in the cytoplasm of thalamic cells and that these increases were markedly attenuated by pharmacological inhibition or genetic knockdown of thalamic Fgr. These data suggest that the increased Fgr is involved in the activation of both NF-κB and ERK1/2 pathways and subsequent production of TNF-α in the hemorrhagic thalamus, although the detailed mechanisms are still unknown. Whether other cytokines (e.g., IL-1β and nitric oxide) as well as chemokines induced by the activated NF-κB and ERK1/2 pathways mediate the role of Fgr in hemorrhage-induced thalamic pain is unknown and open to further study.

In conclusion, the present study, for the first time to our knowledge provides evidence that pharmacological inhibition or genetic knockdown of thalamic hemorrhage-induced increases in Fgr alleviates thalamic pain without changing basal or acute nociceptive responses and locomotor function. Fgr may be a promising therapeutic target in the clinical management of hemorrhage-induced thalamic pain. However, it is worth noting that Fgr may also be expressed in other tissues and involved in other physiological and pathological functions. Thus, careful attention should be paid to potentially unwanted effects caused by Fgr inhibitors.

## Methods

### Animal preparations.

CD1 male mice (about 7–8 weeks) were purchased from Charles River Laboratories. All mice were housed in animal facilities maintaining a standard 12-hour light/12-hour dark cycle, with free access to standard laboratory water and food particles. All efforts were made to minimize the suffering of animals and reduce the number of animals used. To minimize intra- and interindividual variability in behavioral outcome measurements, animals were acclimated for 1–2 days before behavioral testing. The experimenters/observers were blinded to treatment conditions.

### Hemorrhage-induced thalamic pain model.

Mice were anesthetized with isoflurane (5% induction, 2% maintenance) and placed in a stereotactic framework. Under stereotactic guidance, animals were microinjected with 10 nL Coll IV (MilliporeSigma Co.; 0.01 U, dissolved in saline) (16) or fresh autologous blood (15 μL, taken from a tail vein at the same time) (40) into the VPM and VPL of unilateral thalamus (anterior-posterior-anterior-fontanel 0.82–2.30 mm: posterior 1.30–1.95 mm on the lateral side of the midline, and 3.01–4.25 mm on the ventral side of the skull surface). In the sham-operated group, an equal volume of sterile saline was injected instead. After microinjection, the glass micropipette was kept in position for 10 minutes before it was slowly removed. The surgical area was irrigated with sterile saline and iodophor and closed with a wound clip.

### siRNA preparation and microinjection.

*Fgr* siRNA (catalog number: 4457298; Thermo Fisher Scientific Inc.) and negative control scrambled siRNA (catalog number: 4457287; Thermo Fisher Scientific Inc.) were prepared as described previously (41–43). Briefly, 2 μL of siRNA (160 μM) was first diluted with 1 μL of 20% glucose solution (in distilled H_2_O). After gentle mixing, 1 μL of TurboFect in vivo transfection reagent (Thermo Fisher Scientific Inc.) was added. After incubating for 15–20 minutes at room temperature, 0.5 μL of the diluted siRNA solution was microinjected into the ipsilateral VPM and VPL of the thalami in the same manner as the Coll IV microinjection described above.

### Behavioral tests.

Mechanical tests were performed as described previously (44). In brief, each mouse was placed in a Plexiglas chamber on an elevated screen and habituated for 30 minutes. Two calibrated von Frey filaments (0.07 and 0.4 g; Stoelting Co.) were used to stimulate the hind paw for 1–2 seconds, and stimulation was repeated 10 times at 5-minute intervals between the 2 hind paws. A quick paw withdrawal was considered a positive response. Paw withdrawal response for each of these 10 times was counted as a percentage of response frequency: (number of paw withdrawals/10 trials) × 100%.

Thermal tests were then carried out 1 hour later as reported previously (44–46). Briefly, the model 336 Analgesia Meter (IITC Inc.) was used to measure paw withdrawal latency in response to heat. Each mouse was placed in a Plexiglas chamber on a glass plate. A beam of light emitted from the light box was applied to the middle of the plantar surface of each hind paw. A rapid lift of the paw turned off the light. The duration of the beam was defined as the paw withdrawal latency. For each side, 5 trials were conducted with intervals of 5 minutes. A cutoff time of 20 seconds was used to avoid tissue damage.

Finally, the measurement of paw withdrawal latency to noxious cold (0°C) was performed as described previously (44–46). In brief, each mouse was placed in a Plexiglas chamber on a cold aluminum plate with continuous temperature monitoring by a thermometer. Time to mouse jumping was defined as the paw withdrawal latency. Each trial was repeated 3 times at 10-minute intervals. A cutoff time of 20 seconds was used to avoid tissue damage.

Before mice were euthanized, locomotor function tests including placing, grasping, and righting reflexes were performed as described previously (44–46). For the placing reflex, the hind limbs were placed slightly lower than the forelimbs, and the dorsal surface of the hind paws was brought into contact with the edge of the table. Then, whether the hind paws were placed on the table surface reflexively was recorded. For the grasping reflex, mice were placed on the wire grid and then whether the hind paws grasped the wire was recorded. For the righting reflex, the mouse’s back was placed on a flat surface, and whether the mouse would immediately return to a normal upright position was recorded. Each test was repeated 5 times at 5-minute intervals, and scores were recorded by calculating the frequency of each normal response.

### Histological localization of injection sites.

Mice were anesthetized with isoflurane and perfused with 50–100 mL of 4% paraformaldehyde in 0.1 M phosphate-buffered saline (PBS, pH 7.4). Brains were collected, postfixed overnight at 4°C, and cryoprotected in 30% sucrose-containing 0.1 M PBS for 2 days. After brains were sectioned with a thickness of 30 μm, the sections were stained with cresyl violet (Nissl staining) as described previously (16, 47). The location and size of the damaged areas were identified by optical microscopy (Leica DMI4000), and images were analyzed using the NIH ImageJ software package.

### Western blotting assay.

Western blotting was carried out according to our previously published protocol (45, 46). Briefly, the tissues from the injected thalamus were homogenized with ice-cold lysis buffer (10 mM Tris, 5 mM EGTA, 0.5% Triton X-100, 2 mM benzamidine, 0.1 mM phenylmethylsulfonyl fluoride, 40 μM leupeptin, 150 mM NaCl). The crude homogenate was centrifuged at 4°C for 15 minutes at 1000*g*. The supernatants were collected for cytoplasmic protein detection. The pellets were further sonicated and dissolved in nucleus-soluble ice-cold buffer (1 M Tris-HCl, 1% SDS, and 0.1% Triton X-100). After the protein concentration was measured, the sample was heated for 5 minutes at 99°C and loaded onto a 4%–20% precast polyacrylamide gel (Bio-Rad Laboratories). The proteins were electrophoretically transferred onto a PVDF membrane (Bio-Rad Laboratories). The membrane was blocked with 5% skim milk in Tris-buffered saline containing 0.1% Tween-20 for 1 hour, then incubated overnight with the following primary antibodies: goat anti-Fgr (1:500; Abcam; catalog number: SAB2500396), rabbit anti-Src (1:1000; Cell Signaling Technology Inc. [CST Inc.]; catalog number: 2109T), rabbit anti-Lyn (1:1000; CST Inc.; catalog number: 2796T), rabbit anti–TNF-α (1:1000; CST Inc.; catalog number: 6945S), rabbit anti–p-ERK1/2 (1:1000; CST Inc.; catalog number: 4370S), rabbit anti–total ERK1/2 (1:1000; CST Inc.; catalog number: 4695S), rabbit anti–p-NF-κB p65 (1:1000; CST Inc.; catalog number: 3033S), rabbit anti–NF-κB p65 (1:1000; CST Inc.; catalog number: 8242S), mouse anti-GFAP (1:1000; CST Inc.; catalog number: 3670S), rabbit anti-Iba1 (1:500; FUJIFILM Wako Chemicals; catalog number: 019-19741), rabbit anti-GAPDH (1:3000; Santa Cruz Biotechnology Inc.; catalog number: sc-20357), and rabbit anti–histone H3 (1:1000; CST Inc.; catalog number: 9728S). The proteins were detected by goat peroxidase-conjugated anti-mouse or anti-rabbit secondary antibodies (1:3000; Jackson ImmunoResearch Laboratories Inc.; catalog numbers: 145553 for anti-mouse and 146887 for anti-rabbit) or a donkey peroxidase-conjugated anti-goat (1:2000; Jackson ImmunoResearch Laboratories Inc.; catalog number: 147415), developed by western peroxide and luminol/enhancer reagents (Clarity Western ECL Substrate, Bio-Rad Laboratories), and visualized using the ChemiDoc XRS System with Image Lab software (Bio-Rad Laboratories). The intensity of blots was quantified with densitometry using Image Lab software (Bio-Rad Laboratories).

### Total RNA preparation and quantitative real-time reverse transcription PCR.

RNA extraction and real-time reverse transcription PCR assays were carried out as described previously (48). Briefly, total RNA was extracted by the TRIzol method (Invitrogen, Thermo Fisher Scientific Inc.), treated with DNase (New England Biolabs), and reverse-transcribed using ThermoScript reverse transcriptase (Invitrogen, Thermo Fisher Scientific Inc.) and oligo (dT) primers (Invitrogen, Thermo Fisher Scientific Inc.). Templates (1 μL) were amplified by real-time PCR using primers for *Fgr* (forward: 5′-CTCCCCAGAGTAGGTAGTAGGG-3′; reverse: 5′-GTGCCGGAAACCTGGTCTCAA-3′) and *Gapdh* (forward: 5′-TGTTCCTACCCCCAATGTGT-3′; reverse: 5′-TGTGAGGGAGATGCTCAGTG-3′). Each sample was run in triplicate in a 20 μL reaction with 250 nM forward and reverse primers, 10 μL of SsoAdvanced Universal SYBR Green Supermix (Bio-Rad Laboratories), and 20 ng of cDNA. PCR reactions were performed with an initial 3-minute incubation at 95°C, followed by 40 cycles at 95°C for 10 seconds, 60°C for 30 seconds, and 72°C for 30 seconds in a Bio-Rad CFX96 real-time PCR system. Ratios of mRNA levels from different time points to mRNA level from the naive group (day 0) were calculated by using the ΔCt method (2^-ΔΔCt^). All data were normalized to *Gapdh*, an internal control that exhibits stable expression even after hemorrhagic insults (49, 50).

### Immunofluorescence.

After mice were deeply anesthetized with isoflurane, they were perfused with 50–100 mL of 4% paraformaldehyde in 0.1 M phosphate buffer (pH 7.4). Brains were harvested, postfixed at 4°C for 24 hours, and cryoprotected in 30% sucrose overnight. The tissues were sectioned at 30 μm thickness using a cryostat (Leica). After blocking with PBS containing 5% goat serum and 0.3% Triton X-100 for 1 hour at 37°C, the sections were incubated overnight at 4°C with mouse anti-Fgr (1:50; Santa Cruz Biotechnology Inc.; catalog number: sc-74542) alone; with a mixture of mouse anti-Fgr (1:50) and rabbit anti-NeuN (1:500; Invitrogen, Thermo Fisher Scientific Inc. catalog number: 702022), rabbit anti-GFAP (1:500; MilliporeSigma Co.; catalog number: SAB5600060), rabbit anti-Iba1 (1:1000; FUJIFILM Wako Chemicals; catalog number: 019-19741), or rabbit anti-CD68 (1:800; Abcam; catalog number: SAB5500070); with a mixture of mouse anti-Fgr (1:50), rabbit anti–NF-κB p65 (1:400; CST Inc.; catalog number: 8242S), and goat anti-Iba1 (1:500; Invitrogen, Thermo Fisher Scientific Inc.; catalog number: PA5-18519); with a mixture of mouse anti-Fgr (1:50), rat anti–TNF-α (1:500; Invitrogen, Thermo Fisher Scientific Inc.: catalog number: 14-7321-81) and rabbit anti-Iba1 (1:1000), with a mixture of rabbit anti–NF-κB p65 (1:500), rat anti–TNF-α (1:500; Invitrogen, Thermo Fisher Scientific Inc.; catalog number: 14-7321-81); and rabbit anti-Iba1 (1:1000); or with a mixture of rabbit anti-ERK1/2 (1:500), mouse anti-Fgr (1:50), and goat anti-Iba1 (1:500). The sections were then incubated for 1 hour at room temperature with donkey anti–mouse IgG conjugated with Cy2 (1:500; Jackson ImmunoResearch Laboratories Inc.; catalog number: 715-225-151) alone; a mixture of donkey anti–mouse IgG conjugated with Cy2 (1:500; Jackson ImmunoResearch Laboratories Inc.; catalog number: 715-225-151) and goat anti–rabbit IgG conjugated with Cy3 (1:500; Jackson ImmunoResearch Laboratories Inc.; catalog number: 115-035-003); or a mixture of goat anti–rabbit IgG conjugated with DyLight 405 (1:500; Jackson ImmunoResearch Laboratories Inc.), Cy2 (1:500), donkey anti–rat or anti–mouse IgG conjugated with Cy3 (1:500), and donkey anti–goat IgG conjugated with Cy2 (1:500). Control experiments included omission of the primary antiserum and substitution of normal mouse, rabbit, or goat serum for the primary antiserum. The sections were finally mounted using VectaMount permanent mounting medium (Vector Laboratories, Maravai LifeSciences) or VECTASHIELD plus DAPI mounting medium (Vector Laboratories, Maravai LifeSciences). All images were taken using a Leica DMI4000 fluorescence microscope and captured with a DFC365FX camera (Leica). Single-, double- or triple-labeled cells were counted manually or using the NIH ImageJ software.

### Statistics.

Animals were randomly assigned to various treatment groups. All data were expressed as mean ± SEM. Two-tailed, unpaired Student’s *t* tests and 1-way or 2-way ANOVAs with repeated measures were used to analyze the data. When ANOVAs showed significant differences, pairwise comparisons between the means were analyzed by the post hoc Tukey’s method (Sigma Plot 12.5). Significance was set at *P* < 0.05.

### Study approval.

Animal experiments were conducted with the approval of the Institutional Animal Care and Use Committee of Rutgers, The State University of New Jersey. All experiments were conducted in accordance with the ethical guideline *Guide for the Care and Use of Laboratory Animals* (National Academies Press, 2011) of the NIH and the International Association for the Study of Pain.

## Author contributions

YXT conceived the project and supervised all experiments. TH, GF, JG, and YXT designed the project. TH, GF, JG, WC, SW, SJ, and SX carried out molecular and biochemical experiments and performed animal models and behavioral tests. TH, GF, AB, TB, JG, and YXT analyzed the data. TH and JG wrote the manuscript draft. TB, AB, and YXT edited the manuscript. All the authors read and discussed the manuscript.

## Supplementary Material

supplemental data

## Figures and Tables

**Table 1:**
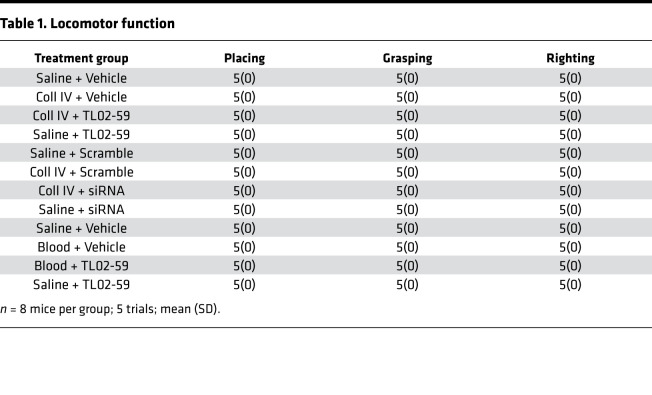
Locomotor function

## Version 1 10/15/2020

Electronic publication

## References

[1] Kumar B, Kalita J, Kumar G, Misra UK (2009). Central poststroke pain: a review of pathophysiology and treatment. Anesth Analg.

[2] Kumar G, Soni CR (2009). Central post-stroke pain: current evidence. J Neurol Sci.

[3] Seifert CL, Mallar Chakravarty M, Sprenger T (2013). The complexities of pain after stroke--a review with a focus on central post-stroke pain. Panminerva Med.

[4] Widar M, Ahlström G (2002). Disability after a stroke and the influence of long-term pain on everyday life. Scand J Caring Sci.

[5] Nandi D, Smith H, Owen S, Joint C, Stein J, Aziz T (2002). Peri-ventricular grey stimulation versus motor cortex stimulation for post stroke neuropathic pain. J Clin Neurosci.

[6] Pickering AE, Thornton SR, Love-Jones SJ, Steeds C, Patel NK (2009). Analgesia in conjunction with normalisation of thermal sensation following deep brain stimulation for central post-stroke pain. Pain.

[7] Mulla SM (2015). Management of central poststroke pain: systematic review of randomized controlled trials. Stroke.

[8] Ellis A, Bennett DL (2013). Neuroinflammation and the generation of neuropathic pain. Br J Anaesth.

[9] Hanada T, Kurihara T, Tokudome M, Tokimura H, Arita K, Miyata A (2014). Development and pharmacological verification of a new mouse model of central post-stroke pain. Neurosci Res.

[10] Hiraga SI, Itokazu T, Hoshiko M, Takaya H, Nishibe M, Yamashita T (2020). Microglial depletion under thalamic hemorrhage ameliorates mechanical allodynia and suppresses aberrant axonal sprouting. JCI Insight.

[11] Nagasaka K, Takashima I, Matsuda K, Higo N (2017). Late-onset hypersensitivity after a lesion in the ventral posterolateral nucleus of the thalamus: a macaque model of central post-stroke pain. Sci Rep.

[12] Ingley E (2008). Src family kinases: regulation of their activities, levels and identification of new pathways. Biochim Biophys Acta.

[13] Kovács M (2014). The Src family kinases Hck, Fgr, and Lyn are critical for the generation of the in vivo inflammatory environment without a direct role in leukocyte recruitment. J Exp Med.

[14] Lowell CA, Fumagalli L, Berton G (1996). Deficiency of Src family kinases p59/61hck and p58c-fgr results in defective adhesion-dependent neutrophil functions. J Cell Biol.

[15] Klebe D (2018). Intracerebral hemorrhage in mice. Methods Mol Biol.

[16] Cai W (2018). Disrupting interaction of PSD-95 with nNOS attenuates hemorrhage-induced thalamic pain. Neuropharmacology.

[17] Kuan YH, Shih HC, Tang SC, Jeng JS, Shyu BC (2015). Targeting P(2)X(7) receptor for the treatment of central post-stroke pain in a rodent model. Neurobiol Dis.

[18] Weir MC (2018). Selective inhibition of the myeloid Src-family kinase Fgr potently suppresses AML cell growth in vitro and in vivo. ACS Chem Biol.

[19] Wasserman JK, Koeberle PD (2009). Development and characterization of a hemorrhagic rat model of central post-stroke pain. Neuroscience.

[20] Huang LN (2019). Fn14 participates in neuropathic pain through NF-κB pathway in primary sensory neurons. Mol Neurobiol.

[21] He L (2020). Toll-like receptor 7 contributes to neuropathic pain by activating NF-κB in primary sensory neurons. Brain Behav Immun.

[22] MacLellan CL (2008). Intracerebral hemorrhage models in rat: comparing collagenase to blood infusion. J Cereb Blood Flow Metab.

[23] Dydyk AM, Munakomi S. Thalamic pain syndrome. In: *StatPearls*. StatPearls Publishing; 2020. 32119377

[24] Vartiainen N (2016). Thalamic pain: anatomical and physiological indices of prediction. Brain.

[25] Lohman AW (2019). Regulation of pannexin channels in the central nervous system by Src family kinases. Neurosci Lett.

[26] Okada M (2012). Regulation of the SRC family kinases by Csk. Int J Biol Sci.

[27] Salter MW, Pitcher GM (2012). Dysregulated Src upregulation of NMDA receptor activity: a common link in chronic pain and schizophrenia. FEBS J.

[28] Kalia LV, Gingrich JR, Salter MW (2004). Src in synaptic transmission and plasticity. Oncogene.

[29] Ge MM (2020). Src-family protein tyrosine kinases: a promising target for treating chronic pain. Biomed Pharmacother.

[30] Keep RF, Zhou N, Xiang J, Andjelkovic AV, Hua Y, Xi G (2014). Vascular disruption and blood-brain barrier dysfunction in intracerebral hemorrhage. Fluids Barriers CNS.

[31] Nadeau CA (2019). Prolonged blood-brain barrier injury occurs after experimental intracerebral hemorrhage and is not acutely associated with additional bleeding. Transl Stroke Res.

[32] Freeze WM (2018). Blood-brain barrier dysfunction in small vessel disease related intracerebral hemorrhage. Front Neurol.

[33] Zhou L (2010). Treatment of cerebral ischemia by disrupting ischemia-induced interaction of nNOS with PSD-95. Nat Med.

[34] Tamiya S, Yoshida Y, Harada S, Nakamoto K, Tokuyama S (2013). Establishment of a central post-stroke pain model using global cerebral ischaemic mice. J Pharm Pharmacol.

[35] Kang OH (2007). Ethyl acetate extract from Angelica Dahuricae Radix inhibits lipopolysaccharide-induced production of nitric oxide, prostaglandin E2 and tumor necrosis factor-alphavia mitogen-activated protein kinases and nuclear factor-kappaB in macrophages. Pharmacol Res.

[36] Hung AL, Lim M, Doshi TL (2017). Targeting cytokines for treatment of neuropathic pain. Scand J Pain.

[37] Chen Y (2016). JAB1 is involved in neuropathic pain by regulating JNK and NF-κB activation after chronic constriction injury. Neurochem Res.

[38] Baumann J (2018). Golgi stress-induced transcriptional changes mediated by MAPK signaling and three ETS transcription factors regulate MCL1 splicing. Mol Biol Cell.

[39] Tsuda M, Ueno H, Kataoka A, Tozaki-Saitoh H, Inoue K (2008). Activation of dorsal horn microglia contributes to diabetes-induced tactile allodynia via extracellular signal-regulated protein kinase signaling. Glia.

[40] Mracsko E, Javidi E, Na SY, Kahn A, Liesz A, Veltkamp R (2014). Leukocyte invasion of the brain after experimental intracerebral hemorrhage in mice. Stroke.

[41] Xu JT (2014). Opioid receptor-triggered spinal mTORC1 activation contributes to morphine tolerance and hyperalgesia. J Clin Invest.

[42] Zhang J (2016). Contribution of the suppressor of variegation 3-9 homolog 1 in dorsal root ganglia and spinal cord dorsal horn to nerve injury-induced nociceptive hypersensitivity. Anesthesiology.

[43] Xu B (2017). Role of microRNA-143 in nerve injury-induced upregulation of Dnmt3a expression in primary sensory neurons. Front Mol Neurosci.

[44] Li Z (2017). The transcription factor C/EBPβ in the dorsal root ganglion contributes to peripheral nerve trauma-induced nociceptive hypersensitivity. Sci Signal.

[45] Zhao JY (2017). DNA methyltransferase DNMT3a contributes to neuropathic pain by repressing Kcna2 in primary afferent neurons. Nat Commun.

[46] Zhao X (2013). A long noncoding RNA contributes to neuropathic pain by silencing Kcna2 in primary afferent neurons. Nat Neurosci.

[47] Liaw WJ (2005). Spinal glutamate uptake is critical for maintaining normal sensory transmission in rat spinal cord. Pain.

[48] Mao Q (2019). DNMT3a-triggered downregulation of K_2p_ 1.1 gene in primary sensory neurons contributes to paclitaxel-induced neuropathic pain. Int J Cancer.

[49] Zhao X (2017). Neutrophil polarization by IL-27 as a therapeutic target for intracerebral hemorrhage. Nat Commun.

[50] Lu A, Tang Y, Ran R, Ardizzone TL, Wagner KR, Sharp FR (2006). Brain genomics of intracerebral hemorrhage. J Cereb Blood Flow Metab.

