# Supplementary material for: Fgr contributes to hemorrhage-induced thalamic pain by activating NF-κB/ERK1/2 pathways
Source: JCI Insight. 2020 Oct 15;5(20):e139987. doi: 10.1172/jci.insight.139987 (PMC7605540; doi:10.1172/jci.insight.139987)
Supplement: supplemental data [file jciinsight-5-139987-s087.pdf]

Supplementary Fig. 1

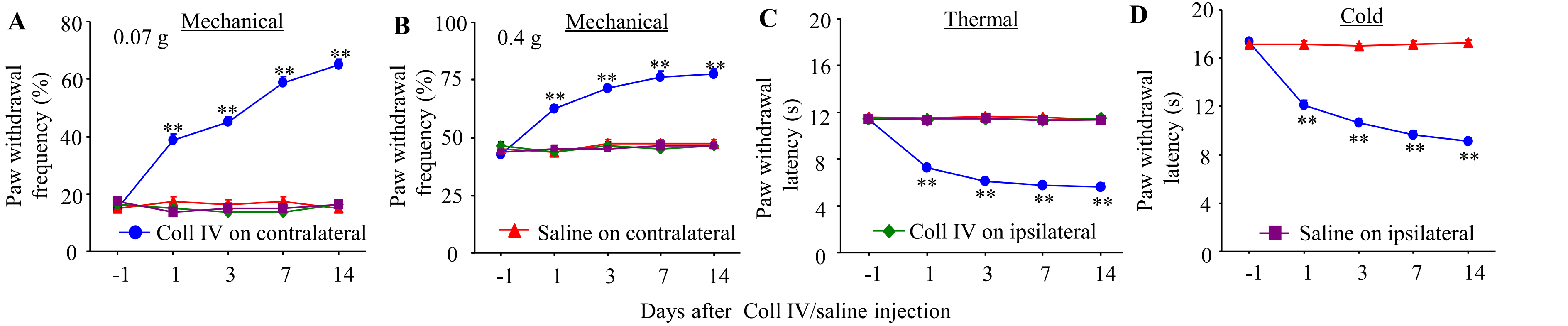

Supplementary Fig. 1. Thalamic microinjection of collagenase IV led to pain hypersensitivities on the contralateral side. (a-d) Microinjection of collagenase IV (Coll IV), but not saline, into the ventral posterior medial nuclei and ventral posterior lateral nuclei of the thalamus led to increased paw withdrawal frequencies in response to 0.07 g (a) and 0.4 g (b) von Frey filaments and to decreased paw withdrawal latencies to heat (c) and cold (d) stimuli on the contralateral side. n = 8 mice/group. Two-way ANOVA with repeated measures followed by *post hoc* Tukey test. $^{**}P < 0.01$ versus the saline group on the contralateral side at the corresponding time point.

Supplementary Fig. 2

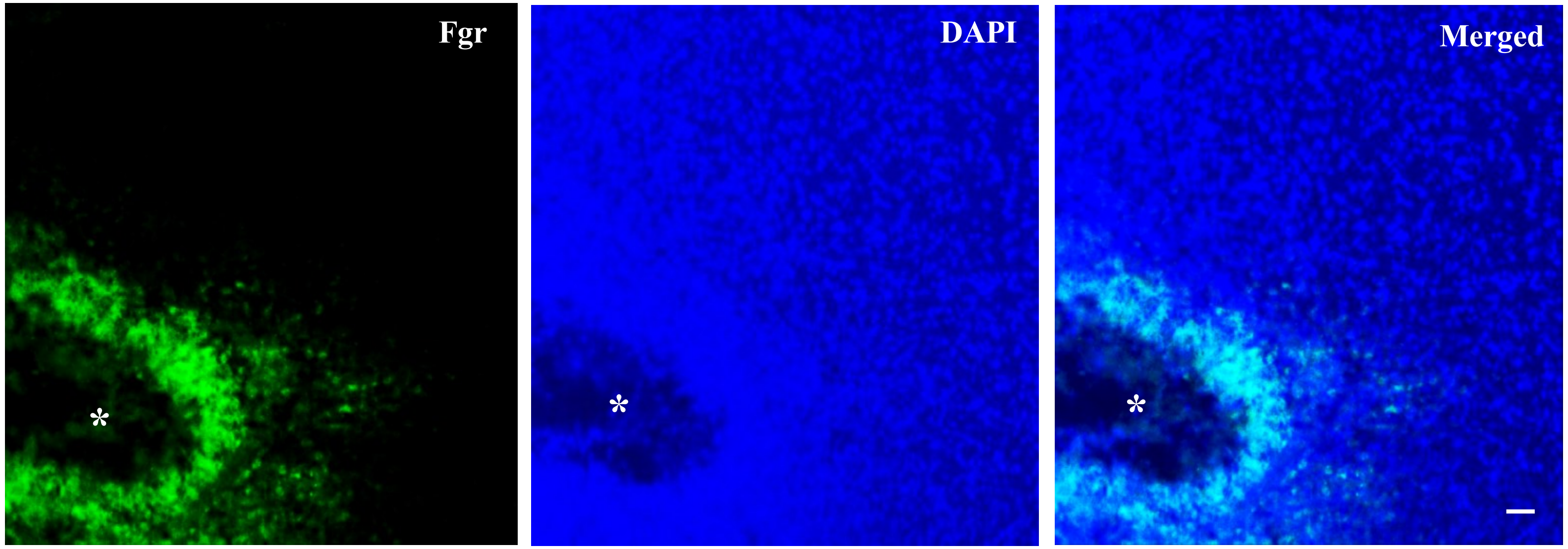

Supplementary Fig.2. Low magnification of representative coronal brain section showing Fgr immunofluoresent stainings of the ipsilateral thalamus on day 7 post-microinjection of Coll IV. Fgr is highly expressed in the regions around/adjacent the cores (*) of hemorrhagic injury in ventral posterior nucleus and ventral posterior mediate nucleus. Scale bar: 100 µm.

Supplementary Fig. 3

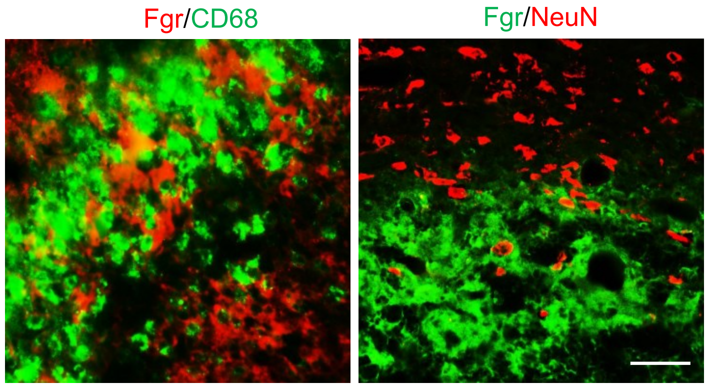

Supplementary Fig. 3. Fgr is not colocalized with CD68 and NeuN in the ipsilateral thalamus on day 3 post-microinjection of Coll IV. Representative samples of n = 3 biological repeats. Scale bar: 100 µm.

Supplementary Fig. 4

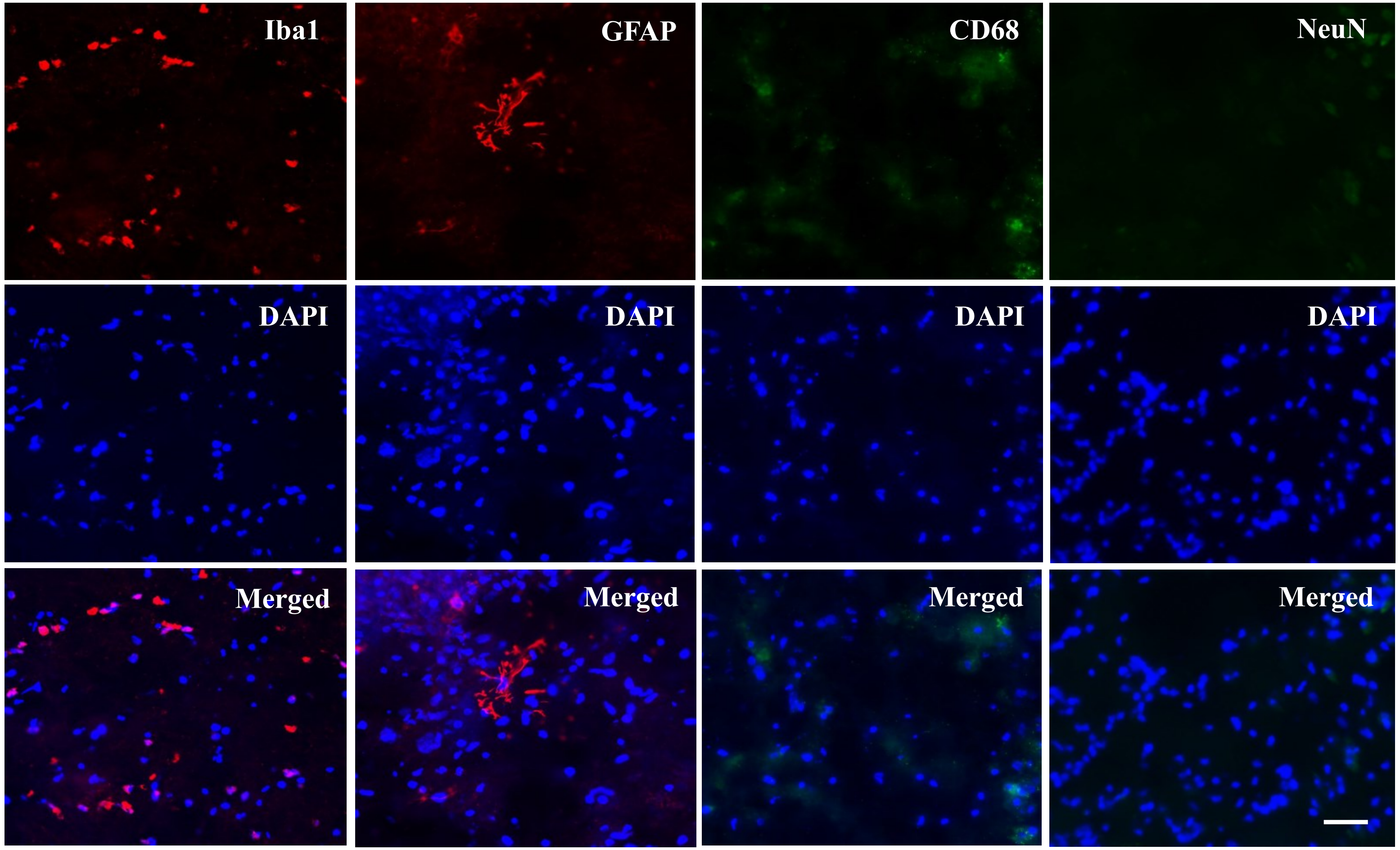

Supplementary Fig. 4. Representative coronal brain sections showing immunofluoresent stainings of Iba1, GFAP, CD68 and NeuN in the core of hemorrhagic injury in ventral posterior nucleus and ventral posterior mediate nucleus on the ipsilateral side on day 7 post-microinjection of Coll IV in the mice with tail vein injection of TL02-59 (15 mg/kg). Scale bar: 100 µm.

Supplementary Fig. 5

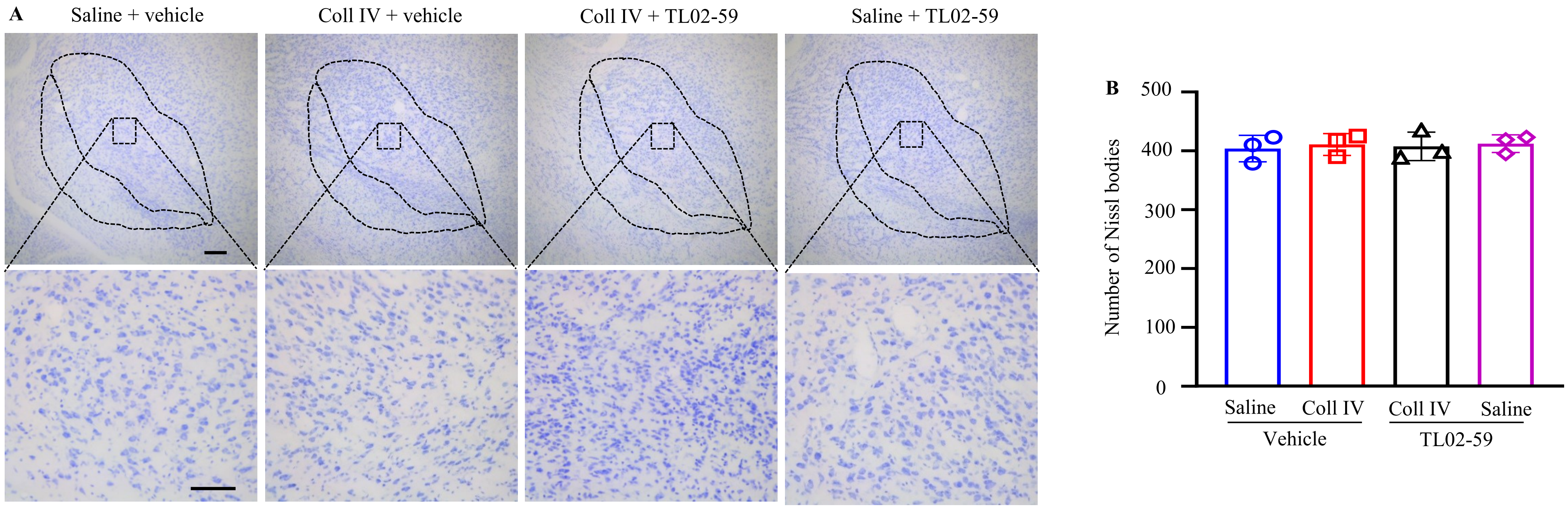

Supplementary Fig. 5. Effect of systemic tail vain administration of TL02-59 (15 mg/kg) or vehicle on number of Nissl-stained cells on the contralateral thalamus on day 3 after thalamic collagenase type IV (Coll IV) or saline. (a) Representative coronal brain sections stained with Nissl from the different treatment. Top: thalamic sections including ventral posterior lateral nucleus (VPL) and ventral posterior medial nucleus (VPM), Scale bar: 100 µm. Bottom: magnification of the corresponding top photographs, Scale bar: 50 µm. (b) Number of Nissl-stained cells in the VPL and VPM of thalami from the different treatment groups as indicated. n = 3 biological repeats/group. Two-way ANOVA with repeated measures followed by *post hoc* Tukey test.

Supplementary Fig. 6

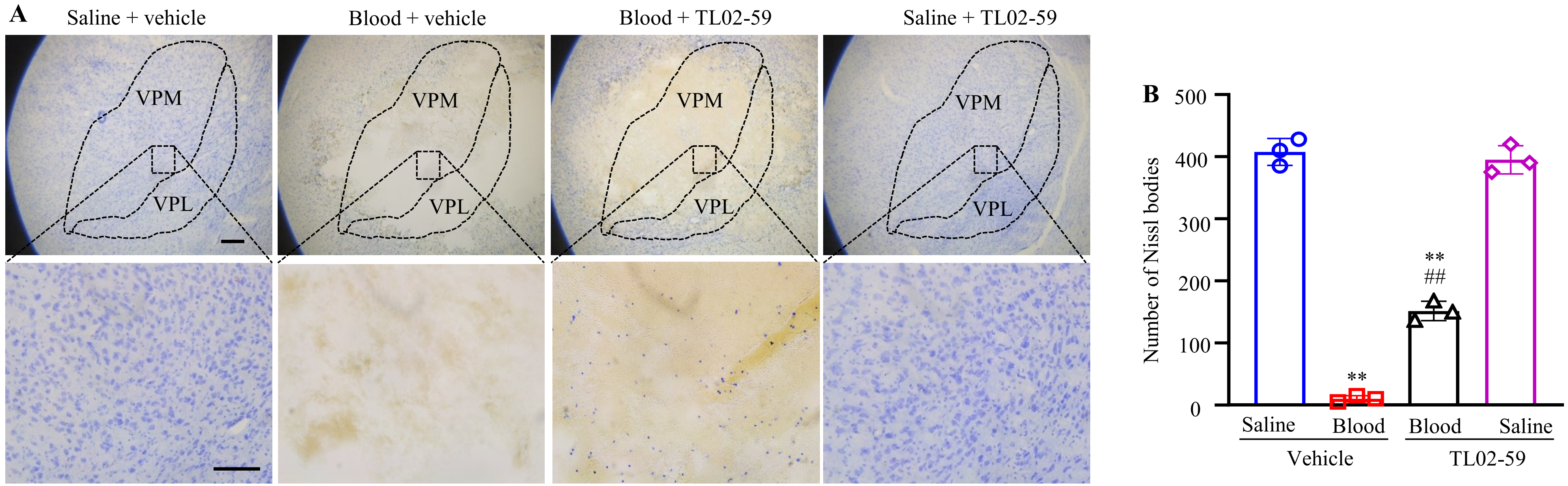

Supplementary Fig. 6. Effect of systemic tail vain administration of TL02-59 (15 mg/kg) on autologous whole blood-caused thalamic lesion. (a): Representative coronal brain sections stained with Nissl from the different treatment groups on day 3 after thalamic microinjection of autologous blood or saline. Top: thalamic sections including ventral posterior lateral nucleus (VPL) and ventral posterior medial nucleus (VPM), scale bar: 100 µm. Bottom: magnification of corresponding top photographs, scale bar: 50 µm. (b) Number of Nissl-stained cells in the VPL and VPM of thalami from the different treatment groups as indicated. n = 3 biological repeats/group. Two-way ANOVA with repeated measures followed by *post hoc* Tukey test. **$P < 0.01$ versus the saline plus vehicle-treated group. $^{\#\#}P < 0.01$ versus the autologous blood plus vehicle-treated group.

Supplementary Fig. 7

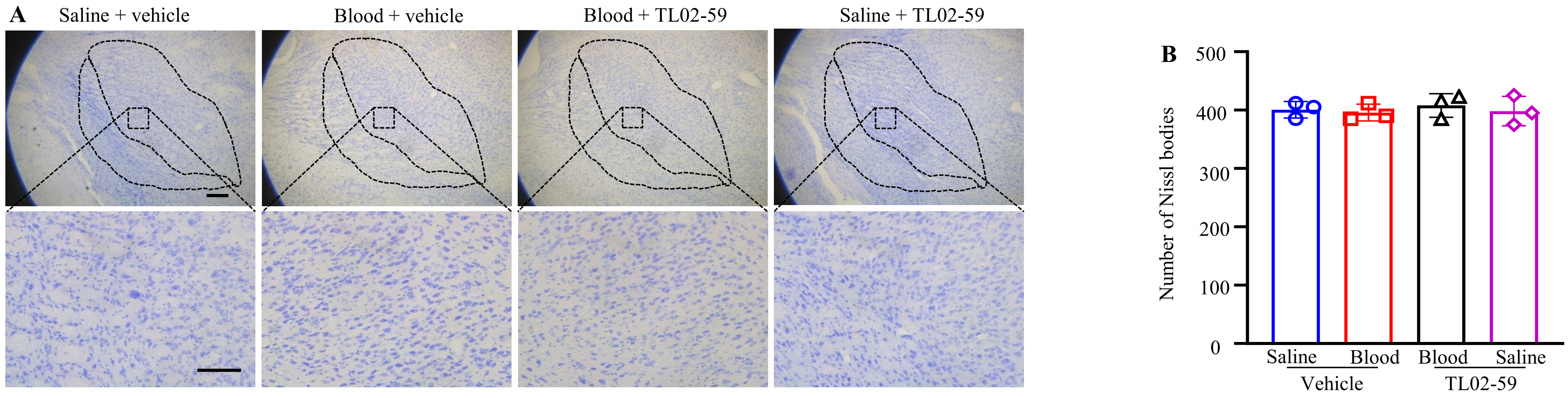

Supplementary Fig. 7. Effect of systemic tail vain administration of TL02-59 (15 mg/kg) or vehicle on number of Nissl-stained cells on the contralateral thalamus on day 3 after thalamic microinjection of autologous blood or saline. (a) Representative coronal brain sections stained with Nissl from the different treatment. Top: thalamic sections includingventral posterior lateral nucleus (VPL) and ventral posterior medial nucleus (VPM), Scale bar: 100 µm. Bottom: magnification of the corresponding top photographs, Scale bar: 50 µm. (b) Number of Nissl-stained cells in the VPL and VPM of thalami from the different treatment groups as indicated. n = 3 biological repeats/group. Two-way ANOVA with repeated measures followed by *post hoc* Tukey test.

Supplementary Fig. 8

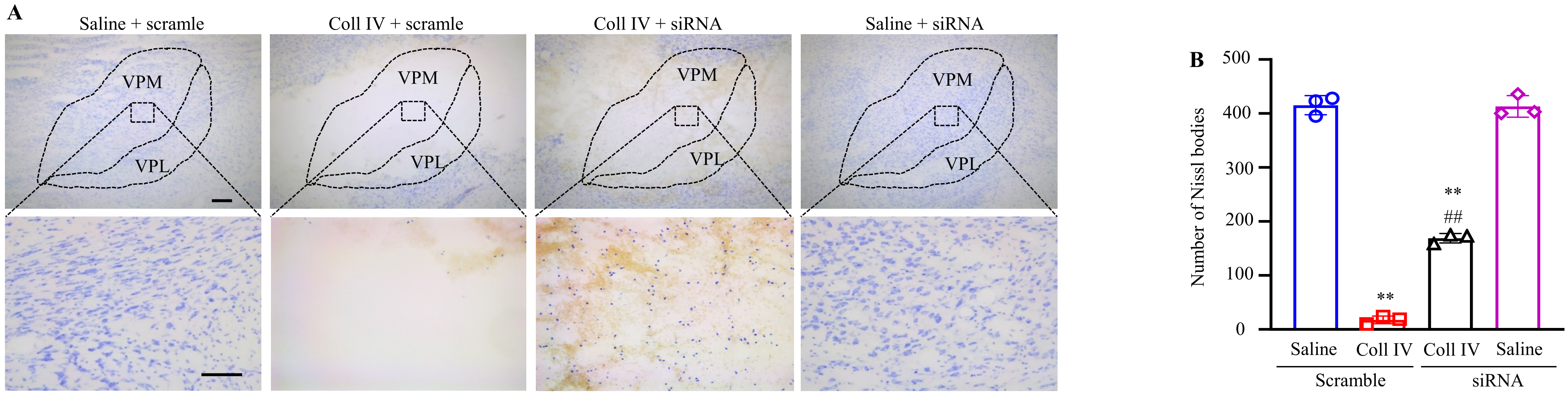

Supplementary Fig. 8. Effect of thalamic microinjection of *Fgr* siRNA on Coll IV microinjection- induced thalamic injury. (a) Representative coronal brain sections stained with Nissl from the different treatment groups on day 5 after thalamic microinjection of collagenase type IV (Coll IV) or saline. Top: thalamic sections including ventral posterior lateral nucleus (VPL) and ventral posterior medial nucleus (VPM), Scale bar: 100 µm. Bottom: magnification of the corresponding top photographs, Scale bar: 50 µm. (b) Number of Nissl-stained cells in the VPL and VPM of thalamus from the different treatment groups. n = 3 biological repeats/group. Two-way ANOVA with repeated measures followed by *post hoc* Tukey test. $^{**}P < 0.01$ versus the saline plus scrambled siRNA-treated group. $^{\#\#}P < 0.01$ versus the Coll IV plus scrambled siRNA-treated group.

Supplementary Fig. 9

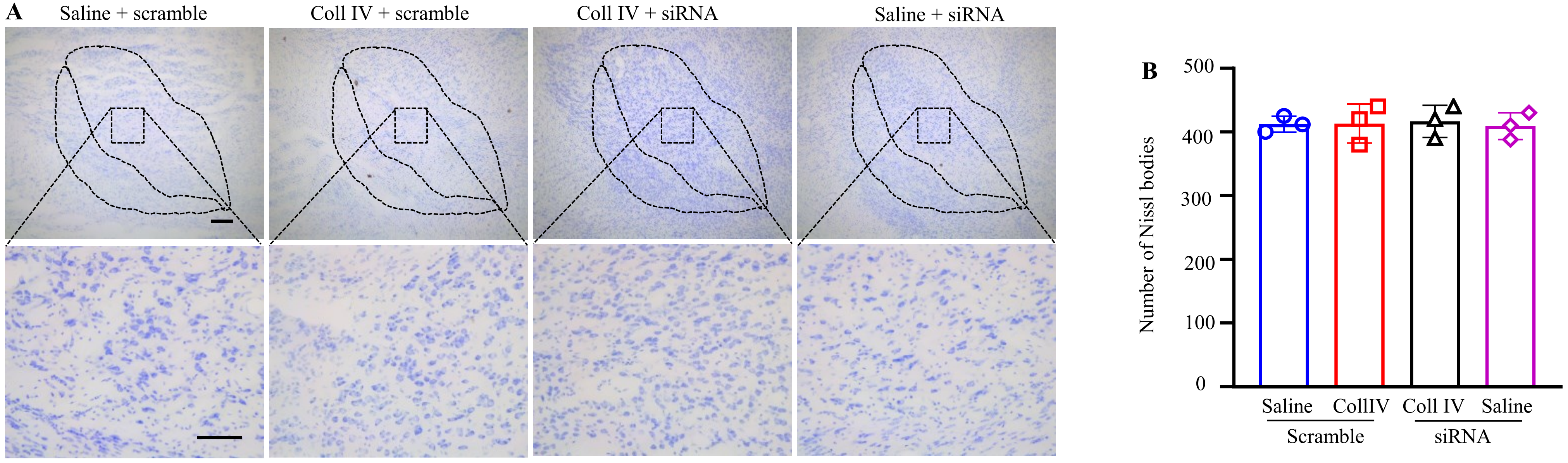

Supplementary Fig. 9. Effect of thalamic microinjection of *Fgr* siRNA or scramble siRNA on number of Nissl-stained cells on the contralateral thalamus on day 5 after thalamic microinjection of collagenase type IV (Coll IV) or saline. (a) Representative coronal brain sections stained with Nissl from the different treatment. Top: thalamic sections including ventral posterior lateral nucleus (VPL) and ventral posterior medial nucleus (VPM), Scale bar: 100 µm. Bottom: magnification of the corresponding top photographs, Scale bar: 50 µm. (b) Number of Nissl-stained cells in the VPL and VPM of thalamus from the different treatment groups as indicated. n = 3 biological repeats/group. Two-way ANOVA with repeated measures followed by *post hoc* Tukey test.
